# Ultrafast charge transfer in mixed-dimensional WO_3-*x*_ nanowire/WSe_2_ heterostructures for attomolar-level molecular sensing

**DOI:** 10.1038/s41467-023-38198-x

**Published:** 2023-05-11

**Authors:** Qian Lv, Junyang Tan, Zhijie Wang, Peng Gu, Haiyun Liu, Lingxiao Yu, Yinping Wei, Lin Gan, Bilu Liu, Jia Li, Feiyu Kang, Hui-Ming Cheng, Qihua Xiong, Ruitao Lv

**Affiliations:** 1 State Key Laboratory of New Ceramics and Fine Processing, School of Materials Science and Engineering, Tsinghua University, Beijing, 100084 China; 2 Shenzhen Geim Graphene Center, Tsinghua-Berkeley Shenzhen Institute and Institute of Materials Research, Shenzhen International Graduate School, Tsinghua University, Shenzhen, 518055 China; 3 Beijing Academy of Quantum Information Sciences, Beijing, 100193 China; 4 State Key Laboratory of Low-Dimensional Quantum Physics and Department of Physics, Tsinghua University, Beijing, 100084 China; 5 Guangdong Provincial Key Laboratory of Thermal Management Engineering and Materials, Tsinghua Shenzhen International Graduate School, Tsinghua University, Shenzhen, 518055 China; 6 Key Laboratory of Advanced Materials (MOE), School of Materials Science and Engineering, Tsinghua University, Beijing, 100084 China; 7 Shenyang National Laboratory for Materials Science, Institute of Metal Research, Chinese Academy of Sciences, Shenyang, 110016 China; 8 Frontier Science Center for Quantum Information, Beijing, 100084 China; 9 Collaborative Innovation Center of Quantum Matter, Beijing, China

**Keywords:** Two-dimensional materials, Optical spectroscopy

## Abstract

Developing efficient noble-metal-free surface-enhanced Raman scattering (SERS) substrates and unveiling the underlying mechanism is crucial for ultrasensitive molecular sensing. Herein, we report a facile synthesis of mixed-dimensional heterostructures via oxygen plasma treatments of two-dimensional (2D) materials. As a proof-of-concept, 1D/2D WO_3-*x*_/WSe_2_ heterostructures with good controllability and reproducibility are synthesized, in which 1D WO_3-x_ nanowire patterns are laterally arranged along the three-fold symmetric directions of 2D WSe_2_. The WO_3-x_/WSe_2_ heterostructures exhibited high molecular sensitivity, with a limit of detection of 5 × 10^−18 ^M and an enhancement factor of 5.0 × 10^11^ for methylene blue molecules, even in mixed solutions. We associate the ultrasensitive performance to the efficient charge transfer induced by the unique structures of 1D WO_3-x_ nanowires and the effective interlayer coupling of the heterostructures. We observed a charge transfer timescale of around 1.0 picosecond via ultrafast transient spectroscopy. Our work provides an alternative strategy for the synthesis of 1D nanostructures from 2D materials and offers insights on the role of ultrafast charge transfer mechanisms in plasmon-free SERS-based molecular sensing.

## Abstract (web summary)

2D materials are promising substrates for surface-enhanced Raman scattering (SERS)-based molecular sensing, but their performance is usually inferior to their plasmonic counterparts. Here, the authors report the synthesis of 1D/2D WO_3-x_/WSe_2_ heterostructures, showing high molecular sensitivity associated to ultrafast charge transfer timescales of ~1 ps.

## Introduction

Ultrasensitive molecular sensing is of great significance in many fields including homeland security, energy and environmental science, clinical diagnosis^1–4^. Compared with the traditional techniques (e.g., electrochemical sensing, mass spectrometry, polymerase chain reaction, etc.), surface-enhanced Raman scattering (SERS) is a fast and non-destructive technique with both high selectivity and sensitivity for the detection of trace amount of molecules or even a single molecule^5^. Noble-metal-based nanomaterials and nanostructures (e.g., Au, Ag), based on the electromagnetic mechanism (EM), offer a wide range of possibilities^6^, nonetheless the limited resources and low surface uniformity constrain their widespread applications. In the past decade, two-dimensional (2D) materials (e.g., graphene, transition metal dichalcogenides (TMDCs)) have been intensively pursued as promising noble-metal-free SERS substrates due to their atomically flat surface, good chemical stability, and tunable electronic structures^7–10^. The SERS effect of 2D materials is usually attributed to the chemical mechanism (CM)^11^, induced from the charge transfer between the substrates and the probe molecules. However, the limits of detection (LODs) of noble-metal-free 2D materials in previous reports are usually in the range of 10^−9^−10^−15^ M^12–18^. Integrating 2D TMDCs with other nanostructures to construct mixed-dimensional heterostructures, such as 2D-1D, might further boost the SERS performance due to the unique electronic structures and physicochemical properties towards enhanced functionalities^19^. In addition, the interactions between such hybrid materials and the analytes often remain elusive and are imperative to be unveiled by time-resolved spectroscopy for the ultrasensitive molecular sensing^20–22^.

At present, the synthesis strategies inevitably hinder the practical applications of TMDCs-based heterostructures in integrated nanoelectronics or sensors^23,24^. For example, aligned transfer can lead to the stacking of 2D or 1D materials into vertical heterostructures with well-designed sequences and desired angles, but it is hard to be scaled up^25^. Chemical vapor deposition (CVD) is a feasible route to synthesize large-area TMDC-based heterostructures, but the controllability and repeatability are severely influenced by many factors^26,27^. In comparison, post treatment is a direct method to prepare heterostructures by selectively converting the target area of TMDCs into foreign phases or foreign components^28^. Lin et al.^29^ directly synthesized MoX (M = Mo or W, X = S or Se) nanowires by steering a focused electron beam, which were connected to the TMDCs monolayer forming 1D-2D heterostructures. However, complex and precise procedures, as well as rigorous conditions with poor scalability, are usually inevitable. Thus, developing a facile and efficient route for the synthesis of mixed-dimensional heterostructures is crucial but still very challenging.

Herein, we propose an oxygen plasma treatment strategy to synthesize mixed-dimensional heterostructures. As a proof-of-concept, 1D/2D WO_3-*x*_/WSe_2_ heterostructures are synthesized by selectively converting the top WSe_2_ layer to WO_3-*x*_ nanowires. The 1D WO_3-*x*_ nanowires are laterally arranged along the three-fold symmetric directions of WSe_2_. The WO_3-*x*_/WSe_2_ heterostructures demonstrate an ultrasensitive SERS effect, which can reach a low LOD of 5 × 10^−18^ M and a high enhancement factor (EF) of 5.0 × 10^11^ for probing methylene blue (MB) molecules. The ultrasensitive SERS capability of heterostructures can be attributed to the efficient charge transfer induced by the unique structures of WO_3-*x*_ nanowires and the effective interlayer coupling of the heterostructures. Furthermore, we unveil that the charge transfer timescale is around 1.0 ps by the transient spectroscopy, illustrating the ultrafast charge transfer process between the heterostructures and the probe molecules.

## Results

### Structural transformation induced by oxygen plasma treatment

**Fig. 1:**
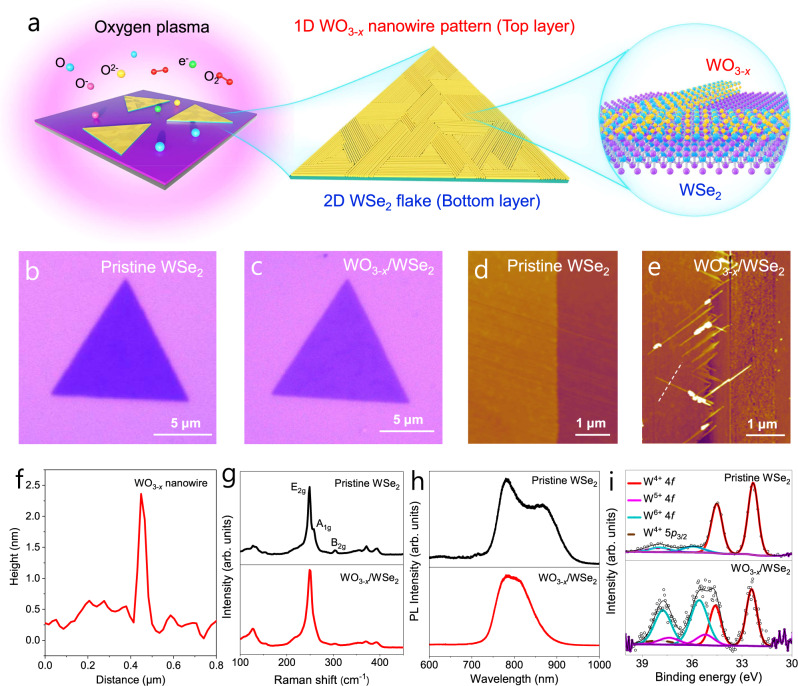
Formation and morphology of 1D/2D WO_3-*x*_/WSe_2_ heterostructures. **a** Schematic illustration of the synthesis of 1D oriented WO_3-*x*_ nanowires from 2D WSe_2_ flake via oxygen plasma treatment. **b**, **c** Optical images of pristine WSe_2_ and WO_3-*x*_/WSe_2_ heterostructures. **d**, **e** Atomic force microscope (AFM) images of pristine WSe_2_ and WO_3-*x*_/WSe_2_ heterostructures. **f** Height profile of an individual WO_3-*x*_ nanowire corresponding to the white dotted line in (**e**). **g** Raman spectra, **h** photoluminescence spectra, and **i** X-ray photoelectron spectroscopy (XPS) fine scan spectra of pristine WSe_2_ and WO_3-*x*_/WSe_2_ heterostructures.

Monolayer and few-layer WSe_2_ flakes were synthesized with high (8:1) and low (8:3) weight ratios of WO_3_ and NaCl, respectively, via an atmospheric-pressure chemical vapor deposition (AP-CVD) route (see Methods and Supplementary Fig. 1). Figure 1a illustrates the structural transformation process. After oxygen plasma treatment, the top WSe_2_ layer can be converted to the preferentially arranged 1D WO_3-*x*_ nanowires, indicated by the inset transmission electron microscopy (TEM) image, which is highly reproducible (Supplementary Figs. 2 and 3). Firstly, we investigated such 2D WSe_2_-to-1D WO_3-*x*_ conversion process on monolayer samples by regulating the plasma treatment durations and found that the 60 s treatment time is enough for the final transformation. (Supplementary Figs. 4–6). Then, 1D/2D WO_3-*x*_/WSe_2_ heterostructures were constructed by exposing the few-layer WSe_2_ to oxygen plasma for 60 s. Optical images show that the contrast of pristine WSe_2_ slightly shallows after the top layer was oxidized to WO_3-*x*_ (Fig. 1b, c), consistent with the phenomena of oxidized monolayer WSe_2_. Pristine WSe_2_ exhibits an atomically flat surface according to the atomic force microscope (AFM) topographic image (Fig. 1d). Interestingly, there are 1D WO_3-*x*_ nanowires stretched from the edge after forming WO_3-*x*_/WSe_2_ heterostructures (Fig. 1e), which are parallel or at an angle of 120° to the edge, following the three-fold symmetry of bottom WSe_2_. The AFM images of heterostructures obtained by different plasma durations (e.g., 15 s, 30 s, 45 s) were also presented, showing similar oriented alignment relationship (Supplementary Fig. 7). According to the statistic length and diameter distributions of WO_3-*x*_ nanowires as the function of the plasma treatment durations (Supplementary Figs. 8 and 9), the lengths and diameters of WO_3-*x*_ nanowires gradually decrease with increasing the treatment durations. This can be explained that prolonging plasma durations could produce more Se vacancies, which would induce more freedom degrees of linear arrangement (see Supplementary Part 10). Thus, Se vacancies will form shorter and narrower linear alignment confined at a flake, leading to the final WO_3-*x*_ nanowires with relatively short lengths and narrow diameters. In addition to treatment duration, the plasma frequency is one key parameter for the formation of WO_3-*x*_ nanowires (Supplementary Fig. 10). The AFM profile indicates that the thickness of an individual WO_3-*x*_ nanowire is nearly 2.0 nm (Fig. 1f).

Raman and photoluminescence (PL) spectra were used to analyze the structures and optical properties of samples. The Raman spectrum of pristine WSe_2_ shows two characteristic peaks of E_2g_ at ~249 cm^−1^ and A_1g_ at ~258 cm^−1^ (Fig. 1g)^30^, respectively. The Raman peak of B_2g_ at ~304 cm^−1^ can be attributed to the interlayer interactions^31^, indicating the nature of few-layer WSe_2_. After forming WO_3-*x*_/WSe_2_ heterostructures, the B_2g_ mode of WSe_2_ is still detected besides E_2g_ and A_1g_, indicating the preservation of the underlying WSe_2_ layers. The pristine WSe_2_ shows prominent PL peaks at ~782 nm and ~860 nm (Fig. 1h), corresponding to the direct and indirect bandgaps of trilayer WSe_2_, respectively. For WO_3-*x*_/WSe_2_ heterostructures, the PL peaks shift to ~779 nm and ~812 nm, which can be assigned to the bilayer emission^32^. According to the X-ray photoelectron spectroscopy (XPS) (Fig. 1i), there are W^6+^ 4*f* and W^5+^ 4*f* chemical states except for the W^4+^ 4*f* peaks in the WO_3-*x*_/WSe_2_ heterostructures compared to that in pristine WSe_2_, which can be attributed to the formation of WO_3-*x*_.

**Fig. 2:**
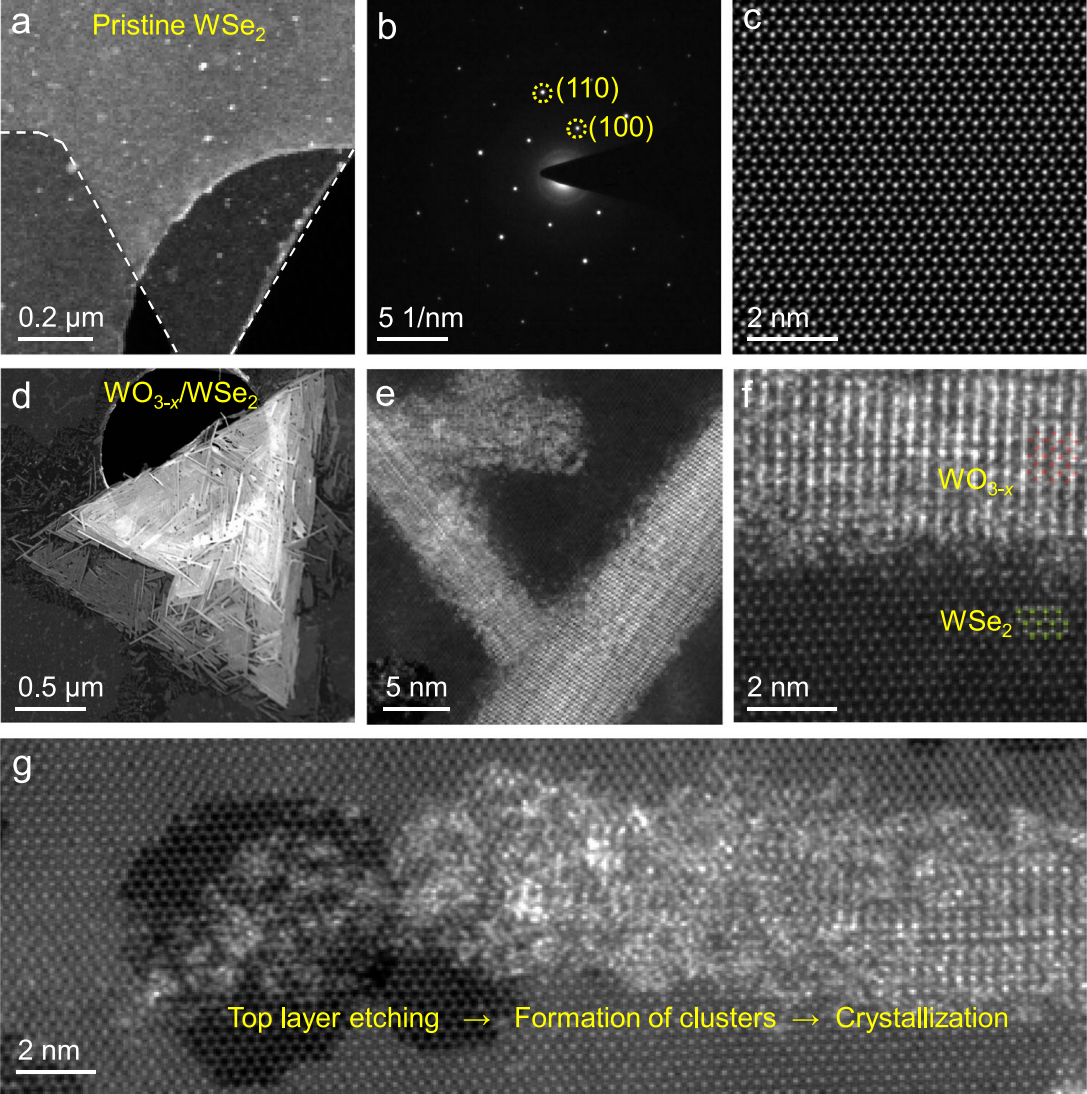
Transmission electron microscope (TEM) characterizations of pristine WSe_2_ and 1D/2D WO_3-*x*_/WSe_2_ heterostructures. **a** TEM image of pristine WSe_2_ supported on a holey carbon-coated TEM grid. **b**, **c** Selected-area electron diffraction (SAED) pattern (**b**) and high-angle annular dark-field scanning TEM (HAADF-STEM) image (**c**) of the pristine WSe_2_. **d**–**f** HAADF-STEM images of WO_3-*x*_/WSe_2_ heterostructures at different magnifications. Gray, green, and red spheres in (**f**) represent W, Se, and O atoms, respectively. **g** HAADF-STEM image of a bilayer WSe_2_ treated by oxygen plasma for only 5 s in order to capture the intermediate states. Partial WSe_2_ was etched and formed amorphous clusters, which were then crystallized into WO_3-*x*_ nanowires under the plasma atmosphere.

The synthesis of WO_3-*x*_ nanowires from pristine WSe_2_ was further investigated by microscopy analysis. Figure 2a shows the morphology of one exposed corner of the triangular-shape WSe_2_, and the corresponding selected-area electron diffraction (SAED) pattern (Fig. 2b) proves its single crystalline nature. The high-angle annular dark-field scanning transmission electron microscopy (HAADF-STEM) image further shows the well-ordered honeycomb structure of pristine WSe_2_ (Fig. 2c). In comparison, for the oxygen plasma-treated few-layer WSe_2_, the top WSe_2_ layer is oxidized to 1D WO_3-*x*_ nanowires and formed a woven structure (Fig. 2d). These nanowires are closely arranged, consistent with our AFM results. As evidenced by the SAED pattern (Supplementary Fig. 11a), the underlying WSe_2_ layers still reserve the pristine form during the plasma treatment^33^, where two sets of diffraction spots corresponding to the underlying WSe_2_ (yellow dotted circle) as well as the top WO_3-*x*_ (green dotted circle) can be also observed. Additionally, the crystalline structure of the underlying WSe_2_ was verified to keep the pristine form after immersing the WO_3-*x*_/WSe_2_ heterostructures into 1 M KOH etchant to remove the top WO_3-*x*_ nanowires (Supplementary Figs. 13–16). The 1D WO_3-*x*_ nanowires are preferentially and energetically favorable to arrange along the three-fold symmetric directions of the underlying WSe_2_ (Fig. 2e). Such preferential distribution of WO_3-*x*_ nanowires can also be clearly identified based on the TEM and energy dispersive X-ray spectroscopy (EDS) elemental mapping results observations (Supplementary Figs. 11b, c and 12). Moreover, Fig. 2f shows the interface microstructure of WO_3-*x*_/WSe_2_ heterostructures at atomic scale. Considering that the lattice mismatch at the interfaces of WO_3-*x*_ nanowires can locally introduce strain in order to maintain the thermodynamic stability of the three-fold symmetric alignment (Supplementary Fig. 17a), here we use geometric phase analysis (GPA) to map the strain field at the junction interface (Supplementary Fig. 17b–f). Such lattice distortion can significantly alter the local electronic structures, which might influence the charge transfer process in SERS^34^. All these results demonstrate that the oxygen plasma treatment is self-limited in nature, inducing the formation of 1D/2D WO_3-*x*_/WSe_2_ heterostructures.

In order to further investigate the structural transformation process of 2D WSe_2_-to-1D WO_3-*x*_ nanowires, few-layer 2D WSe_2_ was treated by oxygen plasma only for seconds for observations (Supplementary Fig. 18). At the start of the treatment, top WSe_2_ layer was etched into notches with triangular-like and hexagonal-like shapes, forming separated amorphous clusters (Supplementary Fig. 19). As the reaction continued, these separated clusters are reconstructed into crystalline WO_3-*x*_ nanowires through a crystallization process (Fig. 2g). It is worth mentioning that for these few-layer WSe_2_ samples, the layer numbers of WSe_2_ around WO_3-*x*_ nanowires are usually one layer thinner than that of parent materials (Supplementary Fig. 20), which proves the etching effect of oxygen plasma. Also, there are numbers of amorphous clusters attached at the edge or end of the as-formed WO_3-*x*_ nanowires, presenting an intermediate state of the transformation due to the short treatment time (Fig. 2g). Density functional theory (DFT) calculations were conducted to illustrate this structural transformation process (See details in Supplementary Figs. 21–26). Thus, the structural transformation process of 2D WSe_2_-to-1D WO_3-*x*_ nanowires are well elucidated both in experiment and theory.

### Molecular sensing performance based on SERS effect

**Fig. 3:**
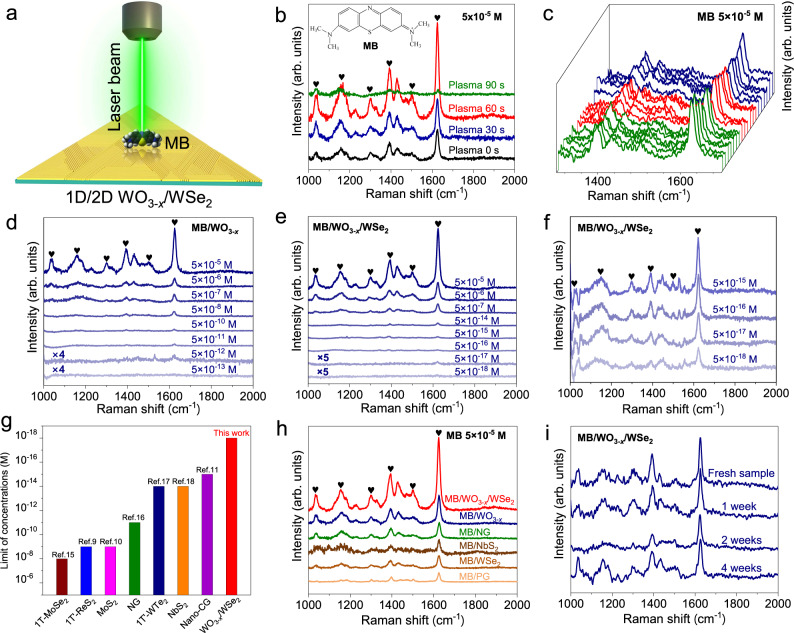
Surface-enhanced Raman scattering (SERS) performance of 1D/2D WO_3-*x*_/WSe_2_ heterostructures. **a** Schematic illustration of Methylene Blue (MB) molecules on WO_3-*x*_/WSe_2_ heterostructures excited by Raman laser beam. **b** Raman spectra of MB on WSe_2_ with different oxygen plasma treatment durations. Inset shows the chemical structure of MB. “♥” symbols represent the characteristic Raman peaks of MB molecules. **c** Raman spectra of MB collected from ~20 spots on WO_3-*x*_ nanowires. **d**–**f** Raman spectra of MB on WO_3-*x*_ nanowires (**d**) and WO_3-*x*_/WSe_2_ heterostructures (**e**, **f**) with different concentrations. **g** Limit of detections (LODs) comparison of the state-of-the-art 2D materials: 1T-MoSe_2_^15^, 1T’-ReS_2_^9^, 1H-MoS_2_^10^, nitrogen-doped graphene (NG)^16^, 1T’-WTe_2_^17^, NbS_2_^18^, and nanocorrugated graphene (Nano-CG)^11^. **h** SERS effects of MB on various 2D materials. **i** Raman spectra of MB on WO_3-*x*_/WSe_2_ heterostructures after exposure in ambient conditions for different durations. Except that the integration time of Raman spectra in (**f**) is 40 s, the integration times for other Raman spectra are all 5 s. WO_3-*x*_ refers to WO_3-*x*_ nanowire patterns unless stated.

Figure 3a illustrates the Raman scattering process of MB molecules on WO_3-*x*_/WSe_2_ heterostructures excited by 633 nm laser. As control experiment, the Raman signals collected from the bare SiO_2_/Si and pristine WSe_2_ are shown in Supplementary Fig. 27. Raman signals of MB molecules on pristine WSe_2_ (marked by “♥”) show stronger intensity than those on bare SiO_2_/Si substrate, indicating the obvious SERS effect of WSe_2_. Figure 3b shows the Raman spectra of MB molecules on monolayer WSe_2_ treated by different oxygen plasma durations and the Raman intensity at ~1620 cm^−1^ are shown in Supplementary Fig. 28. The strongest Raman signals of MB molecules can be obtained when WSe_2_ was completely converted to 1D WO_3-*x*_ nanowires after 60 s plasma treatment. Raman spectra taken from ~20 random points were collected to evaluate the uniformity of MB molecules on WO_3-*x*_ (Fig. 3c), which are nearly identical with a small relative standard deviation (RSD, 16%, Supplementary Fig. 29), indicating the uniform SERS effect of WO_3-*x*_ and the homogeneous adsorption of MB molecules on WO_3-x_. While for the Au nanoparticles (Au NPs) substrates synthesized using a standard sodium citrate reduction method^35^, the Raman intensity of MB molecules at various sites shows huge differences (RSD 72%, Supplementary Fig. 30).

**Fig. 4:**
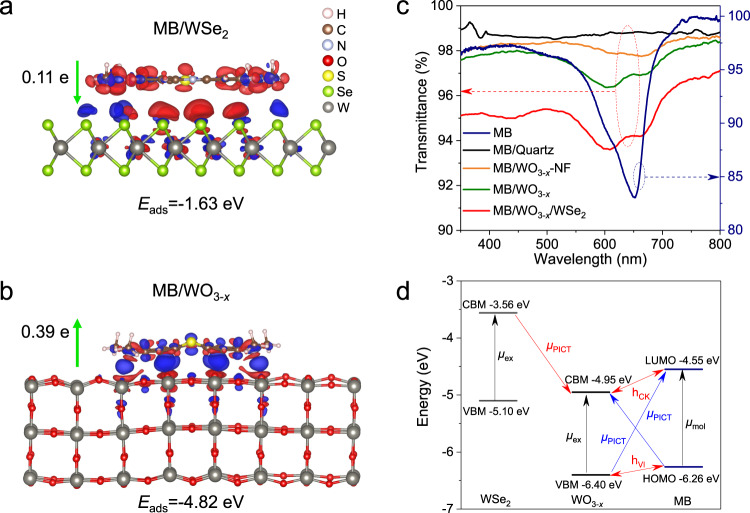
Chemical enhancement mechanism of molecular sensing. **a**, **b** Charge density differences of MB molecules on the WSe_2_ (**a**) and WO_3-*x*_ (**b**). Blue (red) corresponds to the charge accumulation (depletion). *E*_ads_ represents the adsorption energy for MB adsorbed on sample substrates. The isosurface values of MB adsorbed on WSe_2_ and WO_3-*x*_ are 0.00005 and 0.0003 e/A^3^, respectively. **c** Ultraviolet (UV)-visible transmission spectrophotometry of MB/quartz, MB/WO_3-*x*_ nanoflakes (MB/WO_3-*x*_-NF), MB/WO_3-*x*_ and MB/WO_3-*x*_/WSe_2_. **d** Energy level alignment and photo-induced charge transfer (PICT) transition in the MB/WO_3-*x*_/WSe_2_. VBM: valence band maximum. CBM: conduction band minimum. HOMO: highest occupied molecular orbital. LUMO: lowest unoccupied molecular orbital. *μ*_ex_: exciton transition. *μ*_mol_: Molecular transition. *μ*_PICT_: PICT transition. h_CK_, h_VI_: Herzberg-Teller coupling constant.

The LOD of SERS substrates is a vital indicator to evaluate the sensitivity. Therefore, a series of MB solutions with concentrations from 5 × 10^−6^ to 5 × 10^−18 ^M were prepared. The lowest detection concentration of WSe_2_ for MB molecules is only 5 × 10^−8 ^M (Supplementary Figs. 31–32), while the LOD of MB molecules on WO_3-*x*_ can reach a low level of 5 × 10^−12 ^M (Fig. 3d). Even increasing the integration time from 5 s to 40 s, the Raman fingerprints of MB molecules with the concentration of 5 × 10^−13 ^M on 1D WO_3-*x*_ cannot be obtained (Supplementary Fig. 33). WO_3-*x*_ nanoflakes (WO_3-*x*_-NF) were synthesized based on our previous study to verify the dimensional effect (Supplementary Fig. 34)^36^. The Raman intensity of MB on 1D WO_3-*x*_ is nearly 10 times stronger than that on WO_3-*x*_-NF (Supplementary Fig. 35), demonstrating the effective enhancement of the dimensionality on the SERS effect of WO_3-*x*_ nanowire patterns. The adsorption behaviors of MB molecules on WSe_2_ and WO_3-*x*_ were comparably investigated by DFT calculations to elucidate their SERS effects. As shown in Fig. 4a, b, the adsorption energy for MB molecule on the WO_3-*x*_ substrate is −4.82 eV, which is obviously stronger than that on the WSe_2_ substrate (−1.63 eV). Besides, an efficient charge transfer of 0.39 e between the MB molecule and the WO_3-*x*_ substrate can induce a stronger interfacial dipole of MB/WO_3-*x*_ than that of MB/WSe_2_ (0.11 e). Therefore, the sensitive SERS effect of WO_3-*x*_ can be attributed to the following three aspects: (1) the interfacial lattice distortion of the WO_3-*x*_ nanowire patterns will facilitate the charge transfer between substrate and molecules^37,38^, which can be demonstrated by the DFT calculations with the increased charge transfer to 0.40 e after applying biaxial strain to the WO_3-*x*_ nanowire (Supplementary Fig. 36a). (2) The 1D nanowire structure of WO_3-*x*_ facilitates the efficient electron transport along the axis directions^39^. (3) The existence of oxygen vacancies in WO_3-*x*_ can also enhance the interactions with probe molecules via vibronic coupling to further enhance the charge transfer efficiency^40^. Therefore, the excellent molecular sensing performance of WO_3-*x*_ nanowire patterns is attributed to the synergistic effect of the above-mentioned factors.

More importantly, the Raman signals of MB molecules on 1D/2D WO_3-*x*_/WSe_2_ heterostructures are still detectable when the MB solution is down to a low concentration of 5 × 10^−18 ^M as shown in Fig. 3e, which is 3–10 orders of magnitude lower than those of the state-of-the-art 2D materials and comparable or even superior to those of noble-metal-based substrates (Fig. 3g, Supplementary Fig. 37 and Supplementary Table 1). For the ultralow concentrations of MB from 5 × 10^−15^ to 5 × 10^−18 ^M, the integration time for each Raman spectrum was prolonged to 40 s to clearly identify the Raman fingerprints of MB in Fig. 3f. The EF of WO_3-*x*_/WSe_2_ was calculated to be 5.0 × 10^11^ (bulk MB as reference, Supplementary Fig. 38). Even in the mixed solution of MB (5 × 10^−18 ^M) and R6G (5 × 10^−10 ^M) with the 1:1 volume ratio, the Raman fingerprints of MB can also be clearly detected (Supplementary Fig. 39), indicating the ultrasensitive SERS effect of WO_3-*x*_/WSe_2_ heterostructures even under the interference of other molecules. While, the LOD of MB on the 2D/2D WO_3-*x*_-NF/WSe_2_ is 5 × 10^−14 ^M (Supplementary Fig. 40), which is inferior to that of 1D/2D WO_3-*x*_/WSe_2_ benefiting from its unique structure of the preferentially arranged 1D nanowires.

The SERS effect of WO_3-*x*_/WSe_2_ heterostructures can also be extended to detect other dye molecules (e.g., rhodamine 6 G (R6G), crystal violet (CV)). The Raman fingerprints of R6G and CV molecules on WO_3-*x*_/WSe_2_ are clearly distinguished (Supplementary Fig. 41a, b), as marked by the symbols of “♣” and “♦”, respectively. And, the LODs of WO_3-*x*_/WSe_2_ for the detection of R6G and CV are superior to most of the reported non-noble-metal substrates (Supplementary Table 1). Even compared with Au NPs substrates, WO_3-*x*_/WSe_2_ heterostructures still exhibit much higher sensitivity (Supplementary Fig. 41c, d). The different LODs for the detection of various dye molecules on WO_3-*x*_/WSe_2_ heterostructures might be due to the different binding energy and charge transfer process between the substrates and the probe molecules, and different photo-induced charge transfer (PICT) process from the band edges of substrates to the affinity levels of probe molecules. Such demonstrated universal SERS effect of WO_3-*x*_/WSe_2_ will endow it promising potentials in many fields, including food safety, chemical analysis, environmental monitoring, etc. Meanwhile, by choosing different laser lines, Raman fingerprints of individual MB and R6G in the mixed solution can be clearly detected on WO_3-*x*_/WSe_2_ (Supplementary Fig. 42), respectively, demonstrating the excellent selectivity of WO_3-*x*_/WSe_2_ substrate.

Ultraviolet (UV)-visible transmission measurements were carried out to investigate the interactions between the MB and different samples (Fig. 4c). Due to the weak adsorption, the absorption peak of MB in the visible range is hardly detected on quartz. In stark contrast, the absorption peak of MB molecules can be obviously observed upon adsorbed on WO_3-*x*_ and WO_3-*x*_/WSe_2_. Meanwhile, the relative intensity and location of MB peaks on WO_3-*x*_ and WO_3-*x*_/WSe_2_ are remarkably changed compared to those of pristine MB, demonstrating the strong adsorption of MB onto WO_3-*x*_ and WO_3-*x*_/WSe_2_, which can further facilitate the efficient electron transition probability rate between them. Moreover, the energy level alignment between MB and WO_3-*x*_/WSe_2_ heterostructures was calculated by DFT (Fig. 4d). For the MB/WO_3-*x*_ system_,_ the highest occupied molecular orbital (HOMO) and lowest unoccupied molecular orbital (LUMO) levels of MB are at −6.26 and −4.55 eV, respectively. The valence band maximum (VBM) and conduction band minimum (CBM) of WO_3-*x*_ are located at −6.40 and −4.95 eV, respectively. The molecular transition (*μ*_mol_ = 1.71 eV) and exciton transition (*μ*_ex_ = 1.45 eV) in the MB/WO_3-*x*_ system can be excited by 633 nm laser to promote the Raman scattering. And, the PICT transition (1.85 eV) from WO_3-*x*_ VBM to MB LUMO are beneficial to the enhancement of the SERS effect due to the charge transfer resonance, which can borrow intensity from the molecular transition and exciton transition through the Herzberg-Teller coupling constant (h_VI_ and h_CK_) to make the probe molecules much more polarized and further increase the Raman scattering cross-section^41^. The molecular transition resonance can be verified by exciting the Raman signals of MB on sample with different laser lines (Supplementary Fig. 43). When excited by 633 nm laser, the MB molecules demonstrate the strongest Raman intensity compared with those by other laser lines (e.g., 532 nm, 785 nm), since 633 nm laser possesses the nearest energy with the energy level difference (1.71 eV) between LUMO and HOMO of MB. Meanwhile, a charge transfer from the CBM of WSe_2_ to the CBM of WO_3-*x*_ can occur (Fig. 4d). In order to elucidate the effect of electron doping on the SERS effect of WO_3-*x*_/WSe_2_ heterostructures, the surface W atoms in WO_3-*x*_ are doped by electrons. The calculated adsorption energy decreases to −5.05 eV, and the charge transfer increases to 0.40 e for the MB on WO_3-*x*_/WSe_2_ heterostructures. Under the synergetic effect of electron doping and biaxial strain, the charge transfer can further increase to 0.42 e (Supplementary Fig. 36b), which evidences that the charge transfer transition from WSe_2_ to WO_3-*x*_ and the strain between WO_3-*x*_ nanowires can synergistically enhance the SERS performance of WO_3-*x*_ after constructing heterostructures with WSe_2_.

**Fig. 5:**
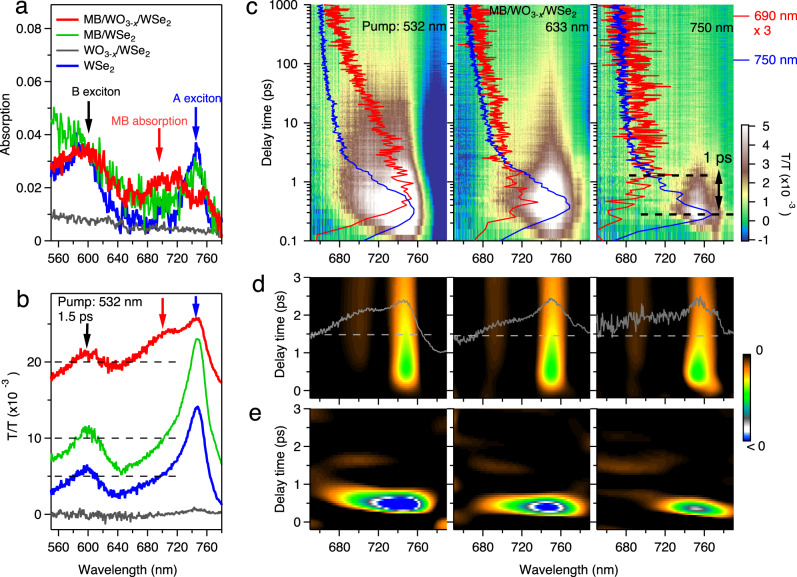
Ultrafast transient absorption/transmission spectra of MB/WO_3-*x*_/WSe_2_. **a** Steady-state optical absorption spectra of MB/WO_3-*x*_/WSe_2_, MB/WSe_2_, WO_3-*x*_/WSe_2_, and WSe_2_. **b** Transient transmission curves of MB/WO_3-*x*_/WSe_2_ at a fixed delay time *t* = 1.5 ps with a pump wavelength of 532 nm. The dashed zero lines are shifted together with the corresponding data curves. **c** Transient transmission images of MB/WO_3-*x*_/WSe_2_ with different pump wavelengths of 532 nm, 633 nm, and 750 nm. The time-dependent curves at 690 nm and 750 nm are in red and blue, respectively. The black arrow depicts the charge transfer timescale of about 1.0 ps. **d**, **e** Secondary derivative images with respect to the wavelength (**d**) and the delay time (**e**), corresponding to the original data in (**c**). The gray solid curves in (**d**) are wavelength-dependent data at 1.5 ps, plotted with the dashed zero lines. Full comparisons are shown in Supplementary Fig. 47.

To accurately quantify the charge transfer process, systematic ultrafast transient absorption/transmission experiments were carried out. In the steady-state spectra (Fig. 5a), the MB absorption spectrum on WO_3-*x*_/WSe_2_ emerges as broadened intensity increase/peak from ~600 to ~700 nm, consistent with the transmission results shown in Fig. 4c, though the light source here is femtosecond (fs) white light continuum (see “Methods”). Meanwhile, the A exciton (750 nm) of WSe_2_ is effectively suppressed, while its B exciton remains unchanged. In Fig. 5b and Supplementary Fig. 47, the transient transmission spectrum of MB/WO_3-*x*_/WSe_2_ shows a pump-induced bleaching peak (positive peak in ΔT/T curves) at ~690 nm, at the expense of the WSe_2_ A exciton. These phenomena have not been found in MB/WSe_2_ or WO_3-*x*_/WSe_2_, suggesting that WO_3-*x*_ plays an intermediary role in the ultrafast charge transfer processes in the form of MB/WO_3-*x*_/WSe_2_. This observation strongly supports our proposal in Fig. 4d. More details in Fig. 5c indicate that the bleaching peak of MB (representative curves at 690 nm) is about 1.0 ps delay after the WSe_2_ A exciton peak, for the case of resonant pumping by 750 nm. We notice that by pumping with 532 and 633 nm, the bleaching curves of MB first present a peak at ~0.5 ps (nearly identical to that of the WSe_2_ A exciton peak due to the thermalization process, i.e., high-energy hot carriers relax to the bottom of conduction bands), followed by a slope change/second peak after 1.0 ps. This can be explained that by over-gap pump, both direct excitation of the bandgap and charge transfer contribute to the pump-induced bleaching feature of MB in different timescales. These dynamics are much more clearly resolved in secondary derivative images shown in Fig. 5d, e (Supplementary Fig. 48). In addition, the transient transmission spectroscopy of the MB adsorbed on 2D/2D WO_3-*x*_-NF/WSe_2_ was carried out for comparison (Supplementary Fig. 49). The MB bleaching signal on WO_3-*x*_-NF/WSe_2_ is nearly an order of magnitude weaker than that on 1D/2D WO_3-*x*_/WSe_2_, suggesting that the 1D/2D WO_3-*x*_/WSe_2_ heterostructures exhibits an enhanced charge transfer process by almost a factor of ten. Overall, the ultrafast spectroscopy results indicate a charge transfer process between WO_3-*x*_/WSe_2_ heterostructures and MB at the timescale of ~1.0 ps.

A series of control experiments were implemented to compare the SERS effects between WO_3-*x*_/WSe_2_ heterostructures and other 2D materials. These 2D materials were synthesized by AP-CVD routes based on our previous works (Supplementary Fig. 44)^16,42^. The strongest Raman intensity of MB molecules can be excited on WO_3-*x*_/WSe_2_ heterostructures (Fig. 3h, Supplementary Fig. 45). The weak Raman signals of MB on NbS_2_ might be ascribed to the adsorption of impurities and the oxidation of metallic NbS_2_ in air. Nitrogen-doped graphene (NG) presents a higher SERS effect than that of pristine graphene (PG), suggesting that defect engineering significantly influences the SERS effect. Furthermore, the stability of WO_3-*x*_/WSe_2_ heterostructures was evaluated (Fig. 3i, Supplementary Fig. 46). Even after exposure in air for a much longer time (4 weeks), there is only a slight degradation (~10% loss) of Raman intensity of MB compared to that on fresh sample. The excellent stability of heterostructures is superior to the metal/semimetal TMDCs^17,18^, which is important for the practical applications. In general, the superior sensitivity, well-resolved Raman fingerprint peaks, excellent selective detection capability, and good stability against the air demonstrate 1D/2D WO_3-*x*_/WSe_2_ heterostructures exhibit promising potentials for the practical applications in the future.

## Discussion

In summary, we demonstrate a concept of 1D nanowire patterns evolved from 2D flakes and their 1D/2D heterostructures by a facile and effective oxygen plasma approach. Take 2D WSe_2_ as an example, as-obtained 1D WO_3-*x*_ nanowires are preferentially and energetically arranged along the three-fold symmetric directions of the parental WSe_2_. An ultrasensitive molecular sensing performance with a low LOD of 5 × 10^−18 ^M and a high EF of 5.0 × 10^11^ for MB molecules is achieved on the WO_3-*x*_/WSe_2_ heterostructures, even in the mixed solution with other molecules. This ultralow LOD of WO_3-*x*_/WSe_2_ heterostructures is 3–10 orders of magnitude lower than those of the state-of-the-art noble-metal-free 2D materials and comparable or even superior to those of noble-metal-based substrates. The charge transfer timescale between the heterostructures and the probe molecules can be clearly unveiled at ~1.0 ps by the ultrafast pump-probe transient spectroscopy. The WO_3-*x*_/WSe_2_ heterostructures also show excellent selectivity, stability, and universal SERS effects for probing other molecules. Our study provides alternative insights into the synthesis of oriented 1D nanowire patterns from 2D materials and prospects for the development of unique mixed-dimensional structures by a facile, effective, and scalable route, which can be applied in diverse fields (e.g., clinical diagnosis, neuromorphic devices, energy storage/conversion). Furthermore, our work also demonstrates the importance of ultrafast transient spectroscopy in SERS fields to unveil the underlying mechanism.

## Methods

### Sample synthesis

Monolayer and few-layer WSe_2_ flakes were grown in the AP-CVD system. WO_3_ powder and Se powder were used as W and Se precursors, respectively. NaCl was added to assist the growth of WSe_2_. The weight ratios of WO_3_ and NaCl were set as 8:1 and 8:3 for the growth of monolayer WSe_2_ and few-layer WSe_2_, respectively. The solution of W precursor was prepared by dissolving WO_3_ powder and NaCl powder into 10 mL ammonia solution under stirring at 80 °C for 1.5 h. Then, a drop of the solution (~5 μL) was spin-coated onto a piece of SiO_2_/Si substrate at 3000 rpm for 60 s. For the growth of WSe_2_, the SiO_2_/Si substrate spin-coated with the precursor film was placed in a quartz boat and loaded into the center of the CVD reactor. Se powder (~800 mg) was placed at another quartz boat and placed at upstream, ~5 cm away from the inlet of the reactor (See Supplementary Fig. 1). Before heating, the quartz tube reactor was purged with ~1500 sccm Ar for ~5 min. The furnace was heated to 850 °C with Ar at a flow rate of 80 sccm, and maintained for 6 min to grow WSe_2_ with Ar/H_2_ (80/6 sccm) flow. Finally, the sample was quickly cooled to the room temperature by pulling the quartz tube out of the furnace.

### Synthesis of 1D WO_3-*x*_ nanowires and 1D/2D WO_3-*x*_/WSe_2_ heterostructures

The lattice reconstruction process of WSe_2_ was carried out in an oxygen plasma system (Schwarze). The oxygen plasma was generated with the frequency of 40 kHz. For the synthesis of 1D WO_3-*x*_ nanowires, monolayer WSe_2_ was placed in the center of the chamber and treated for 2–90 s by oxygen plasma. For the synthesis of heterostructures, few-layer WSe_2_ was treated by oxygen plasma with the same duration as that of monolayer WSe_2_.

### Sample characterization

Optical images were captured by an Olympus BX 53 M microscope. AFM images were recorded using an Oxford MFP-3D Infinity system in a tapping mode. XPS (Thermo Fisher ESCALAB 250Xi) was used to analyze the chemical states. The binding energies were calibrated with C 1 s at 284.8 eV. SEM images were collected on a Zeiss Merlin system. TEM analysis was performed on FEI Tecnai F30 system and JEOL JEM-2100 system, operating at 300 kV and 200 kV accelerating voltages, respectively. HAADF-STEM images were taken on a FEI Spectra 300, operating at 80 kV accelerating voltage. UV-visible transmission measurements were recorded on U-3900H Spectrophotometer at a scan speed of 120 nm min^−1^.

### SERS measurements

MB, R6G, and CV molecules were dissolved in ethanol to form solutions with the initial concentration of 5 × 10^−5 ^M. Then, the dye solutions with different concentrations ranging from 5 × 10^−6^ to 5 × 10^−18 ^M can be prepared by sequential dilution process. Samples grown on SiO_2_/Si substrates (0.5 × 0.5 cm^2^) were immersed into the solutions with different concentrations for ~30 min, respectively, followed by naturally drying. Then, the samples were rinsed with ethanol to remove the free molecules and dried with N_2_ gas. All the Raman spectra were collected in a HORIBA LabRAM HR system with the ×50 objective. The Raman excitation wavelength for probing MB is 633 nm but for probing R6G and CV is 532 nm. The laser power is below 1 mW to avoid the possible heating effect on samples. The integration time for MB, R6G, and CV with the concentrations from 5 × 10^−5^ to 5 × 10^−14^ M is 5 s, while for MB with the concentrations from 5 × 10^−15^ to 5 × 10^−18 ^M is 40 s. The Raman spectra were collected with the same experimental parameters for the better comparison among different 2D materials and noble-metal substrates.

### Ultrafast transient absorption/transmission measurements

The ultrafast pump-probe measurements were conducted in a transient absorption/transmission spectrometer (Helios fire from Ultrafast system) at room temperature. The samples were transferred onto the transparent sapphire substrates. The infrared pulses (800 nm, 35 fs) were provided by a Ti:Sapphire amplifier (Coherent Inc.) working at 1 kHz repetition rate, and splitted into two beams. One went to an optical parameter amplifier (Opera Solo System from Coherent Inc.) to produce tunable pumps (532 nm, 633 nm, and 750 nm), while the other one was delayed by a linear stage and focused into a sapphire crystal to generate white-light continuum as probe. The pump and probe beams were focused on sample surface with spot sizes of about 200 μm and 7 μm, respectively, by applying the parabolic reflectors. All measurements were taken with a constant pump fluence of 38 μJ cm^−2^. A back-thinned CCD linear detector synchronized with an optical chopper was applied to measure the transient transmission difference, through the detection of probe signals with and without pump.

### DFT calculations

All calculations based on density functional theory (DFT) were performed using the Vienna ab initio simulation package (VASP)^43^. The projector augmented wave (PAW) potentials^44^ and generalized gradient approximation (GGA) of the Perdew–Burke–Ernzerhof (PBE) functional^45^ were used for the electron-ion interaction and exchange-correlation energy, respectively. An 8 × 8 × 1 (7 × 4 × 1) supercell for WSe_2_ (WO_3-*x*_) nanosheet was built to investigate the adsorption behaviors of MB molecules. The cutoff energy of the plane wave basis was set to 400 eV. The convergence criteria for the total energy and force were set to 10^−5 ^eV and 0.01 eV Å^−1^, respectively. The vacuum layer of at least 15 Å was chosen to eliminate the interactions between the periodic images. The DFT-D3 method^46^ was used to consider the van der Waals interaction between MB molecules and sample substrates. The adsorption energy for MB adsorbed on sample substrates was calculated using *E*_ads_ = *E*_MB/sample_ − *E*_sample_ − *E*_MB_, where *E*_MB/sample_, *E*_sample_, and *E*_MB_ are the energies of MB adsorbed on sample substrates, sample substrates, and MB molecules, respectively. Because the states near the Fermi level of WO_3-*x*_ were mainly originated from p orbitals of W, the ionic potential method was used to dope electrons on W atoms from W 3d core level to the lowest unoccupied band. This method ensured that the doped electrons were localized around the W atoms and maintained the neutrality of the sample.

## Supplementary information

Supplementary Information

Peer Review File

## Data Availability

The Source data underlying the figures of this study are available at 10.6084/m9.figshare.22565134. All raw data generated during the current study are available from the corresponding authors upon request.
