# Supplementary material for: Ultrafast charge transfer in mixed-dimensional WO3-x nanowire/WSe2 heterostructures for attomolar-level molecular sensing
Source: Nat Commun. 2023 May 11;14:2717. doi: 10.1038/s41467-023-38198-x (PMC10175504; doi:10.1038/s41467-023-38198-x)
Supplement: Supplementary file 1 — Supplementary Information [file 41467_2023_38198_MOESM1_ESM.pdf]

Supplementary Information

Ultrafast charge transfer of mixed-dimensional WO_{3-x} nanowire/WSe_2 heterostructures for attomolar-level molecular sensing

Qian Lv[1,10], Junyang Tan[2,10], Zhijie Wang[2,10], Peng Gu[3,4,10], Haiyun Liu[3], Lingxiao Yu[1], Yinping Wei[2], Lin Gan[2], Bilu Liu[2,*], Jia Li[2,5*], Feiyu Kang[2,5,6], Hui-Ming Cheng[2,7], Qihua Xiong[3,4,8,9*], Ruitao Lv[1,6*]

[1] State Key Laboratory of New Ceramics and Fine Processing, School of Materials Science and Engineering, Tsinghua University, Beijing 100084, China.

[2] Shenzhen Geim Graphene Center, Tsinghua-Berkeley Shenzhen Institute and Institute of Materials Research, Shenzhen International Graduate School, Tsinghua University, Shenzhen 518055, China.

[3] Beijing Academy of Quantum Information Sciences, Beijing 100193, China.

[4] State Key Laboratory of Low-Dimensional Quantum Physics and Department of Physics, Tsinghua University, Beijing 100084, China.

[5] Guangdong Provincial Key Laboratory of Thermal Management Engineering and Materials, Tsinghua Shenzhen International Graduate School, Tsinghua University, Shenzhen 518055, China.

[6] Key Laboratory of Advanced Materials (MOE), School of Materials Science and Engineering, Tsinghua University, Beijing 100084, China.

[7] Shenyang National Laboratory for Materials Science, Institute of Metal Research, Chinese Academy of Sciences, Shenyang 110016, China.

[8] Frontier Science Center for Quantum Information, Beijing 100084, China

[9] Collaborative Innovation Center of Quantum Matter, Beijing 100084, China

[10] These authors contributed equally: Qian Lv, Junyang Tan, Zhijie Wang, Peng Gu.

[*] Corresponding authors. *Email addresses*:
bilu.liu@sz.tsinghua.edu.cn (B. Liu)
li.jia@sz.tsinghua.edu.cn (J. Li)
qihua_xiong@tsinghua.edu.cn (Q. Xiong)
lvruitao@tsinghua.edu.cn (R. Lv)

1. Optical characterizations of WSe_2

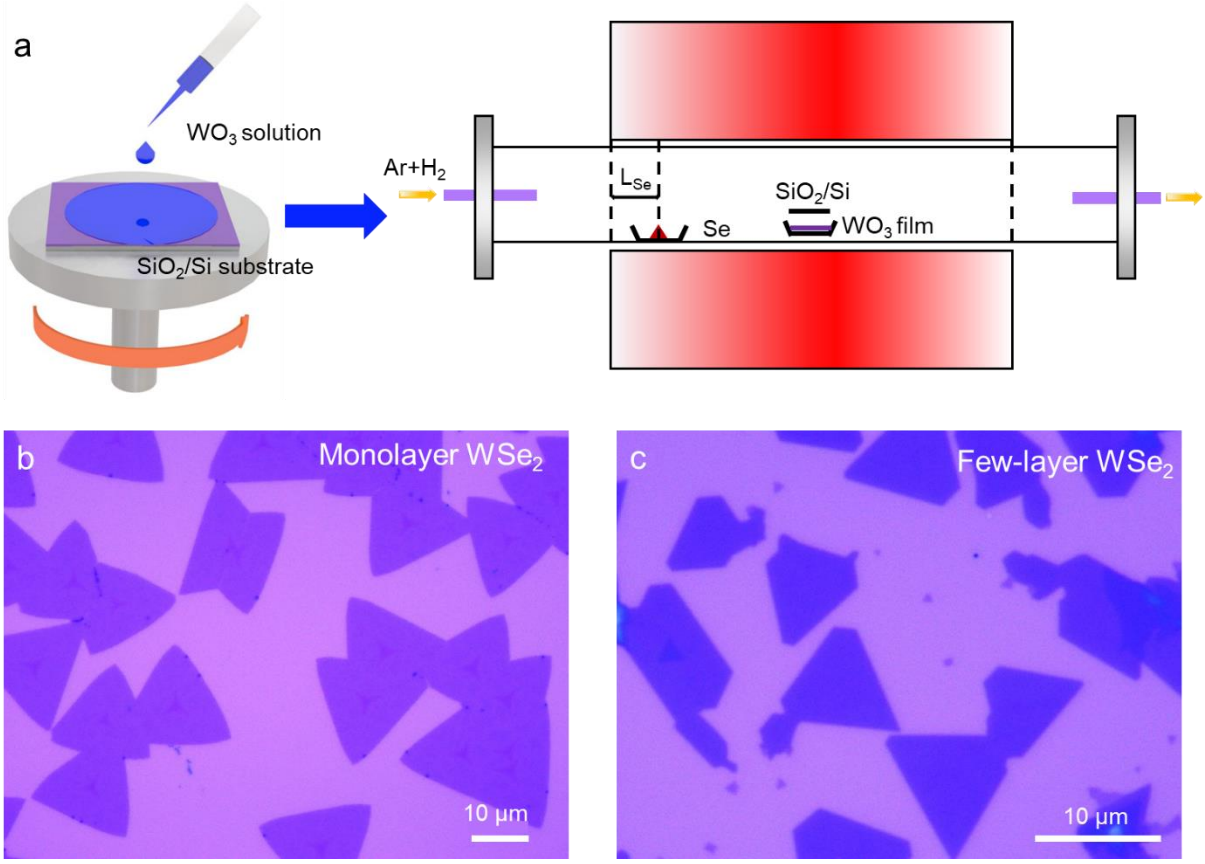

Supplementary Figure 1. (a) Schematic illustration of the growth of two-dimensional (2D) WSe_2 flakes by atmospheric-pressure chemical vapor deposition (AP-CVD) route. The tungsten precursor was prepared by dissolving WO_3 and NaCl powders in ammonia solution and stirring at 80 °C for 1.5 h. Then, a drop of the as-prepared solution was spin-coated on the SiO_2/Si substrate at 3000 rpm for 60 s to form a uniform film. Finally, the SiO_2/Si substrate coated with the precursor film was placed in a CVD quartz reactor for the growth of WSe_2 flakes. More detailed information can be found in **Methods** section. (b-c) Optical images of monolayer and few-layer WSe_2.

2. Reproducibility of 1D/2D WO_{3-x}/WSe_2 heterostructures

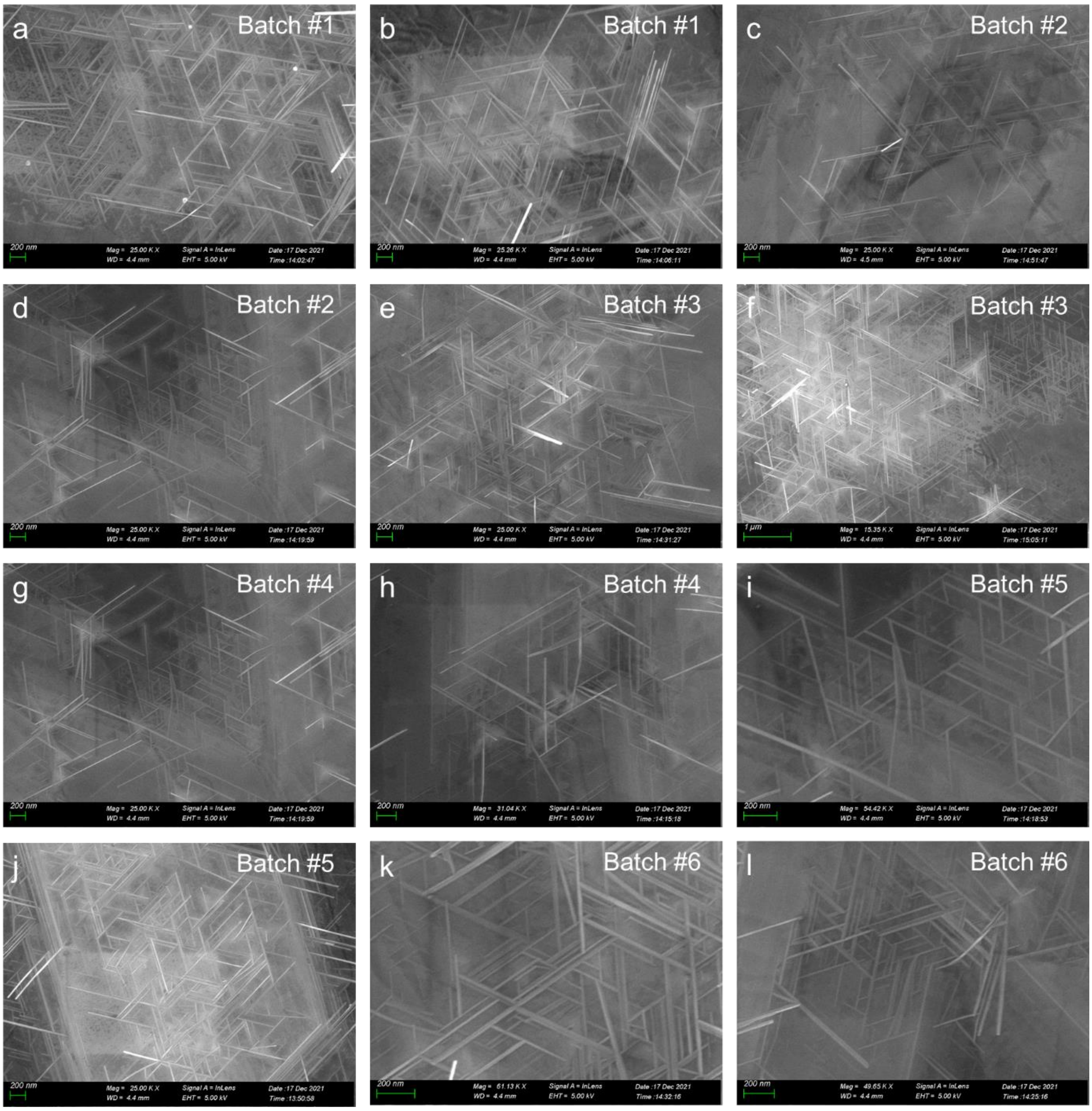

Supplementary Figure 2. Scanning electron microscopy (SEM) images of preferentially oriented 1D WO_{3-x} nanowires on 2D WSe_2 surfaces by treating different batches of CVD-grown WSe_2. (a-b) Batch #1. (c-d) Batch #2. (e-f) Batch #3. (g-h) Batch #4. (i-j) Batch #5. (k-l) Batch #6.

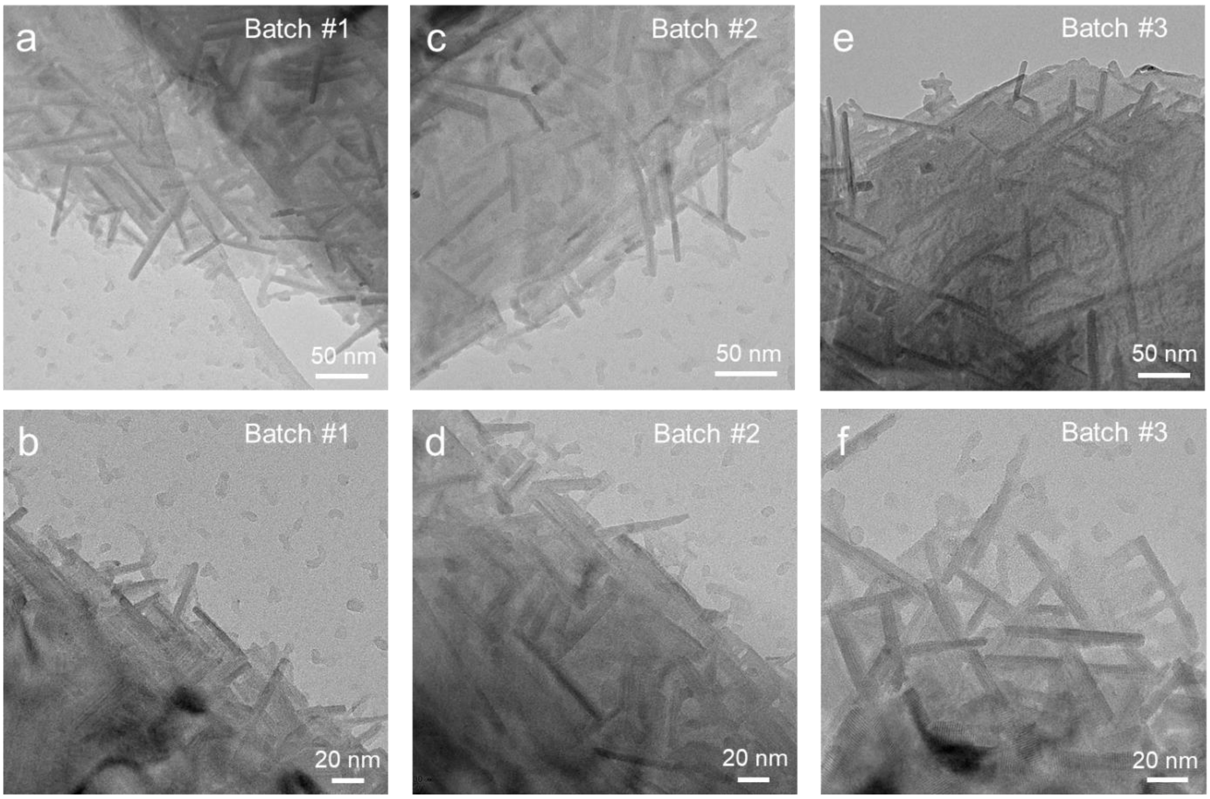

Supplementary Figure 3. Transmission electron microscopy (TEM) images of 1D WO_{3-x} nanowires on different batches of CVD-grown WSe_2 treated by plasma with 40 kHz frequency in another lab's plasma generator (Peking University), which further proves the reproducibility of the conversion process under oxygen plasma. (a-b) Batch #1. (c-d) Batch #2. (e-f) Batch #3.

3. Conversion from monolayer WSe_2 to WO_{3-x}

The morphology of WSe_2 with the oxygen plasma treatment for different durations was characterized by an optical microscope. The optical contrast between WSe_2 flakes and SiO_2 substrates gradually shallows with the increasing of plasma treatment time from 0 s to 90 s (Supplementary Fig. 4), which might be ascribed to the high optical transparency of tungsten oxides[1]. The Raman and photoluminescence (PL) spectra of monolayer WSe_2 disappear with the oxygen plasma treatment for 60 s (Supplementary Fig. 5a-c), suggesting that the monolayer WSe_2 was completely oxidized into WO_{3-x}. It is worth noting that the Raman fingerprint peaks of WO_3 at ~803 cm^{-1} and ~713 cm^{-1} are not observed, which indicates that the tungsten oxide formed on the WSe_2 might be substoichiometric WO_{3-x} (x<3)[2]. X-ray photoelectron spectroscopy (XPS) of monolayer WSe_2 treated by oxygen plasma for 60 s further proves the complete conversion from WSe_2 to WO_{3-x} in terms of the absence of W^{4+} $4f_{7/2}$ and W^{4+} $4f_{5/2}$ peaks located at 32.3 eV and 34.5 eV of WSe_2 (Supplementary Fig. 5d-f). In contrast, the other two pairs of new doublets of W^{6+} $4f$ (35.6 eV and 37.8 eV) and W^{5+} $4f$ (35.2 eV and 37.4 eV) chemical states appear, which can be assigned to the WO_{3-x}[3]. The coexistence of W^{6+} and W^{5+} chemical states also demonstrates the existence of oxygen vacancies in as-obtained tungsten oxide.

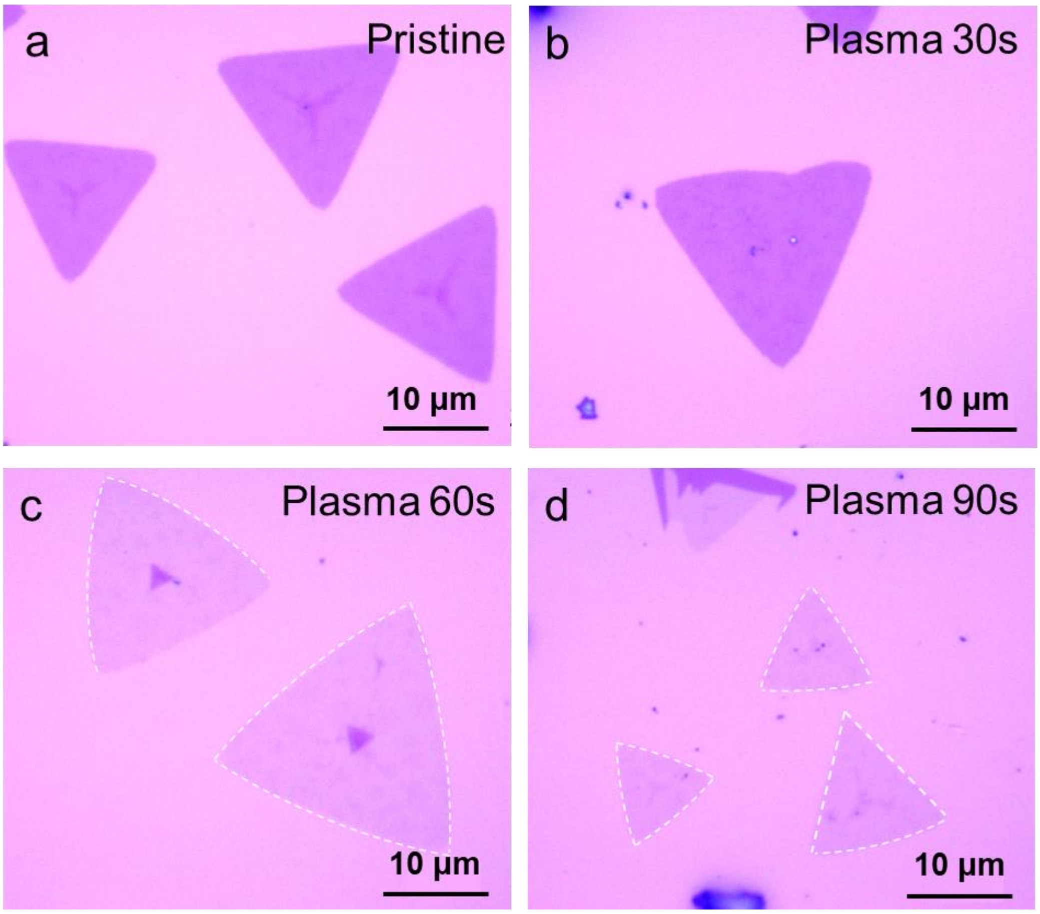

Supplementary Figure 4. Morphology characterizations of 2D WSe_2 flakes before and after plasma treatment. Optical images of as-synthesized WSe_2 with plasma treatment for (a) 0 s, (b) 30 s, (c) 60 s and (d) 90 s.

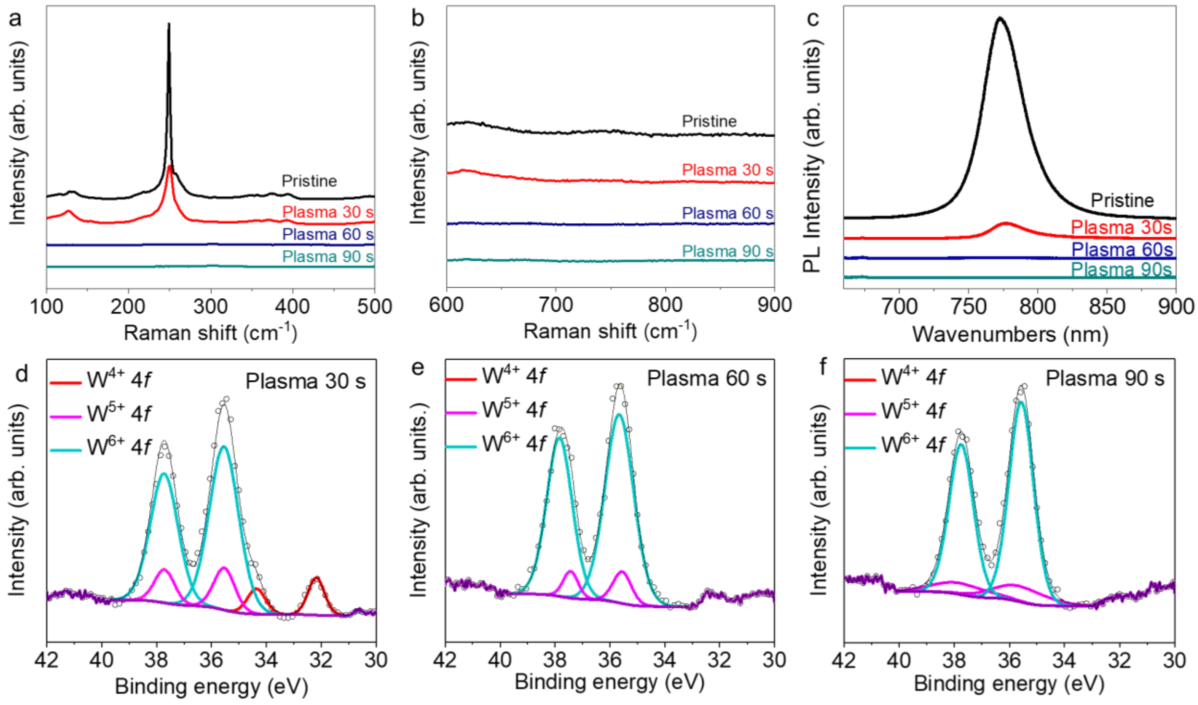

Supplementary Figure 5. (a-b) Raman spectra of monolayer WSe_2 treated by different plasma durations. (c) Photoluminescence (PL) spectra of monolayer WSe_2 treated by different plasma durations. (d-f) W 4*f* fine scan X-ray photoelectron spectroscopy (XPS) of monolayer WSe_2 treated by different plasma durations.

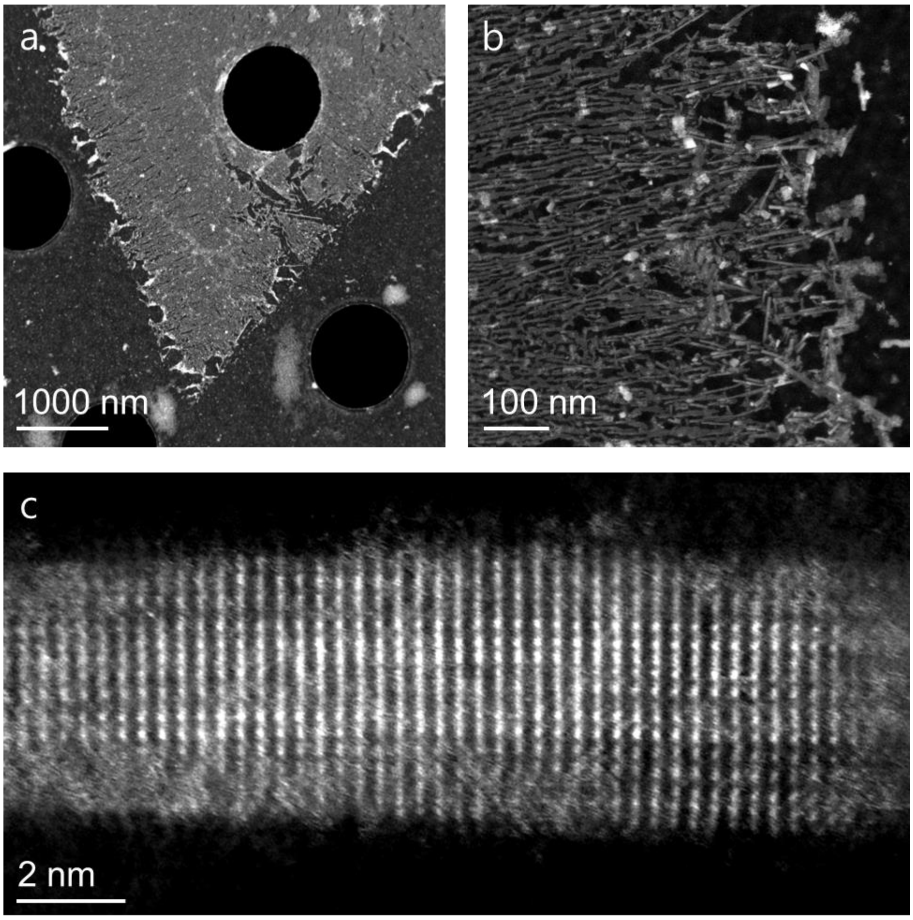

Supplementary Figure 6. High-angle annular dark field scanning TEM (HAADF-STEM) images of the as-obtained oriented 1D nanowires after the complete WSe_2-to-WO_{3-x} conversion from the parent monolayer WSe_2 sample.

4. 2D WSe_2-to-1D WO_{3-x} Evolution

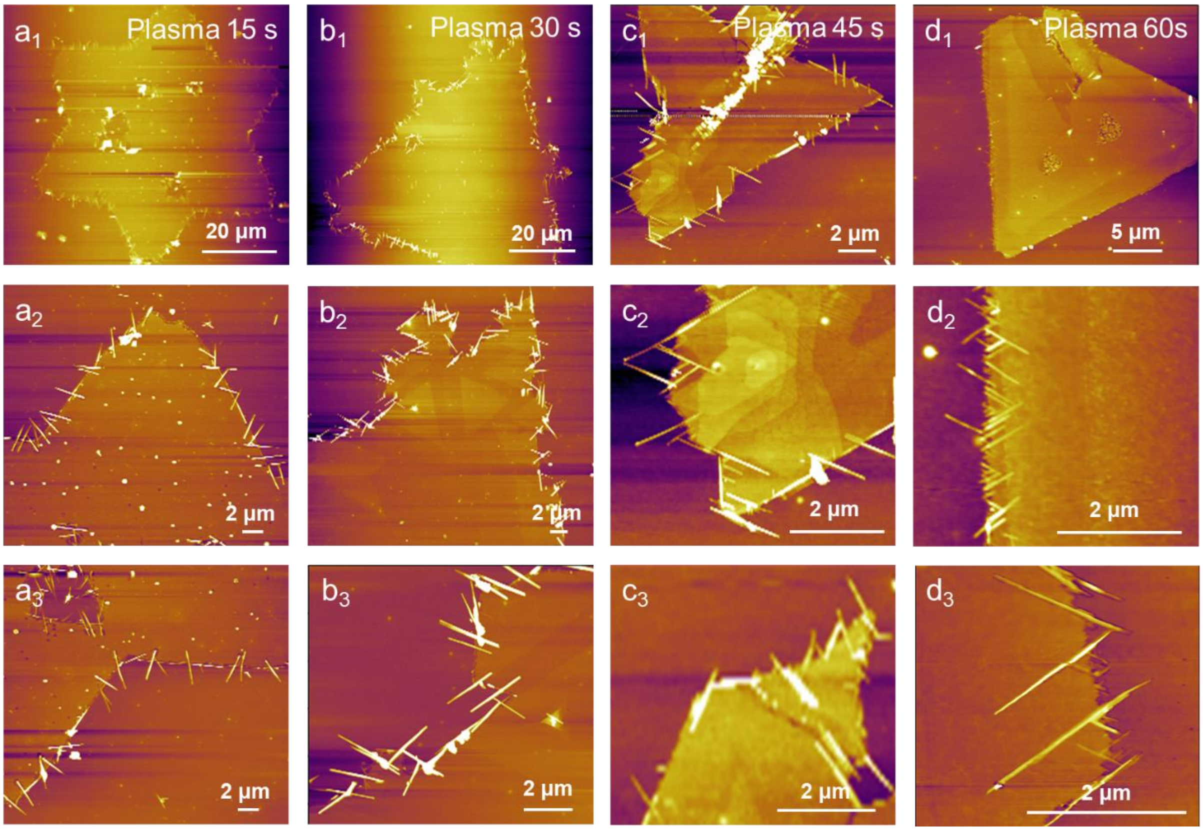

Supplementary Figure 7. Atomic force microscope (AFM) images of WO_{3-x}/WSe_2 heterostructures with different plasma durations. (a_1-a_3) plasma 15 s. (b_1-b_3) plasma 30 s. (c_1-c_3) plasma 45 s. (d_1-d_3) plasma 60 s. The a_2 and a_3 are zoom-in images of a_1, b_2 and b_3 are zoom-in images of b_1, c_2 is a zoom-in image of c_1, and d_2 is a zoom-in image of d_1.

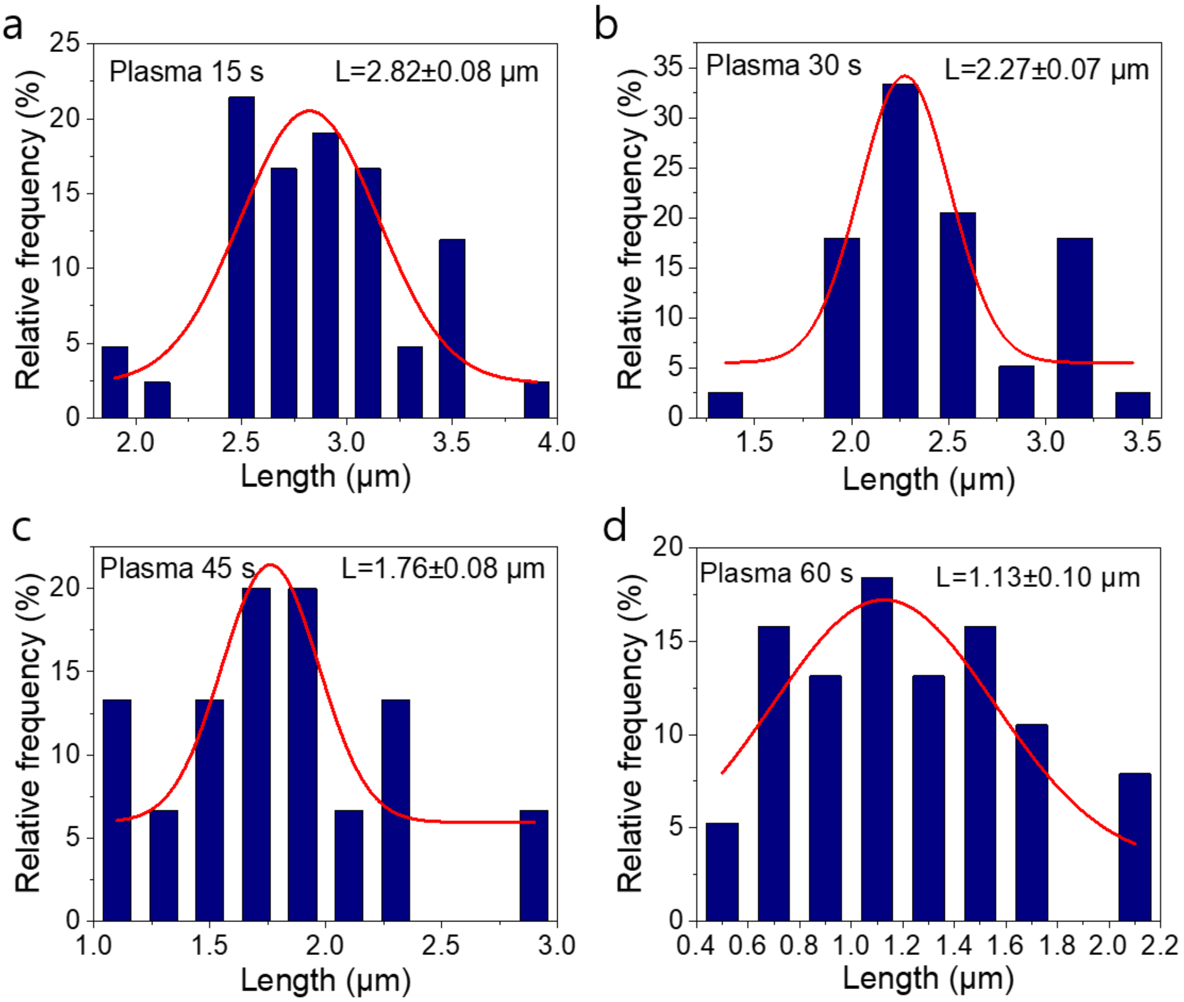

Supplementary Figure 8. Statistic length (L) distributions of WO_{3-x} nanowires as the function of the plasma treatment durations. (a) Plasma 15s. (b) Plasma 30 s. (c) Plasma 45 s. (d) Plasma 60 s. It can be seen that with prolonging the plasma durations, the lengths of WO_{3-x} nanowires gradually decrease.

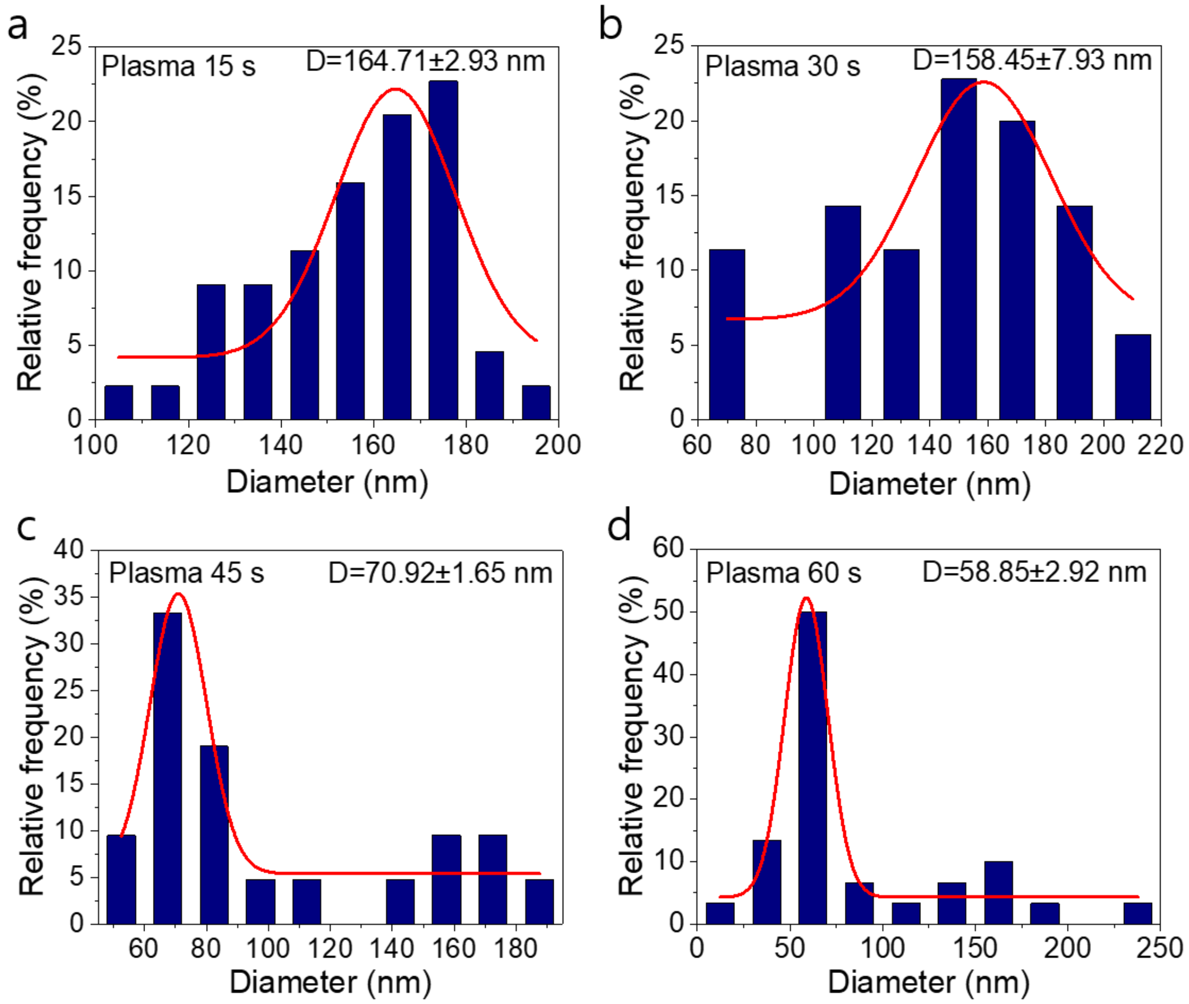

Supplementary Figure 9. Statistic diameter (D) distributions of WO_{3-x} nanowires as the function of the plasma treatment durations. (a) Plasma 15s. (b) Plasma 30 s. (c) Plasma 45 s. (d) Plasma 60 s. It can be seen that with prolonging the plasma durations, the diameters of WO_{3-x} nanowires gradually decrease.

5. Influence of the plasma frequency on the conversion process

In order to verify the influence of plasma frequency, WSe_2 was also treated by the plasma with the frequency of 13.56 MHz. It can be seen from Supplementary Fig. 10 that the surface of WSe_2 cracked after the high-frequency plasma treatment, and no WO_{3-x} nanowires formed. Besides, to better illustrate that the 40 kHz plasma can induce the formation of 1D/2D WO_{3-x}/WSe_2 heterostructures, the different batches of CVD-grown WSe_2 were treated by another plasma generator with the frequency of 40 kHz at the nearly same conditions. As mentioned in Supplementary Fig. 3, the 1D WO_{3-x} nanowires are obtained, which further emphasizes the importance of the plasma frequency on the conversion process.

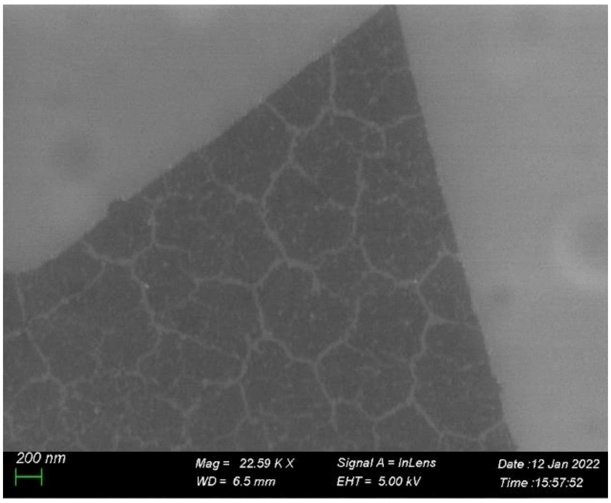

Supplementary Figure 10. SEM image of WSe_2 treated by plasma with 13.56 MHz frequency.

6. Microscopy characterizations of WO_{3-x}/WSe_2 heterostructures

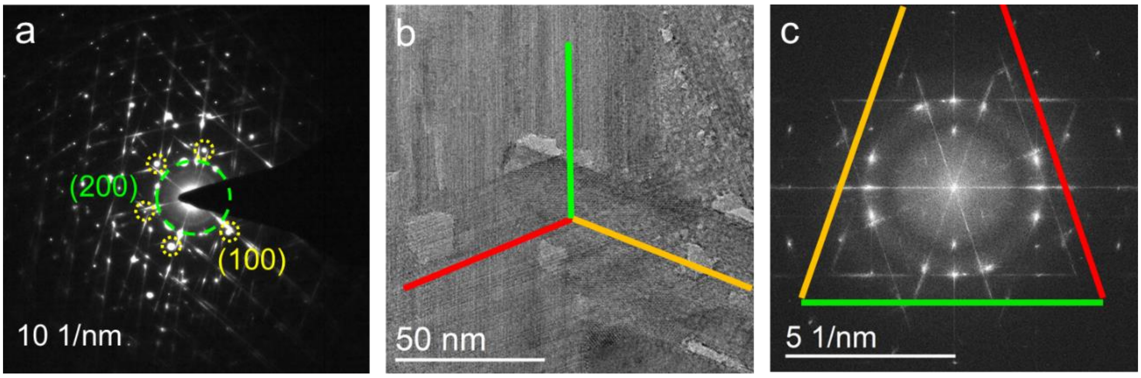

Supplementary Figure 11. (a) Selected-area electron diffraction (SAED) pattern of WO_{3-x}/WSe_2 heterostructures. (b-c) TEM images of the three intersected WO_{3-x} nanowires (b) and the corresponding fast Fourier transform (FFT) image (c). Supplementary Fig. 11b exhibits one typical joint of three WO_{3-x} nanowires, in which the WO_{3-x} nanowires are preferentially and energetically favorable to arrange along the three-fold symmetric directions of the underlying WSe_2. The corresponding FFT pattern in Supplementary Fig. 11c further demonstrates the three-fold symmetric alignment of as-obtained WO_{3-x} nanowires.

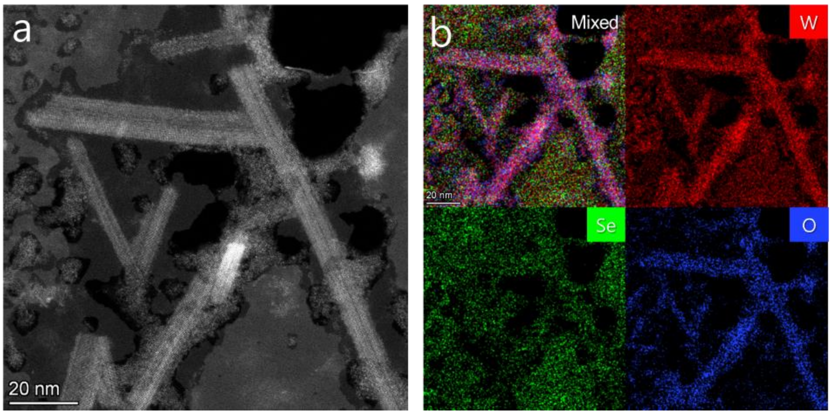

Supplementary Figure 12. (a) Low magnification HAADF-STEM image of WO_{3-x}/WSe_2 heterostructures. (b) Energy-dispersive X-ray spectroscopy (EDS) maps of W, Se, O elements and their overlaid map. It can be clearly observed that the 1D nanowires were made up of W and O elements, while the underlying flake is WSe_2. The 1D WO_{3-x} nanowires are energetically favorable to arrange along the three-fold symmetric directions of the underlyingWSe_2.

7. Characterizations of the underlying WSe_2 in WO_{3-x}/WSe_2 heterostructures

In order to illustrate the crystalline structure of the underlying WSe_2 after plasma treatment, the 1D/2D WO_{3-x}/WSe_2 heterostructures were immersed into 1M KOH etchant for ~5 s to remove the top WO_{3-x} nanowires. As shown in the XPS spectrum in Supplementary Fig. 13, after immersing into 1M KOH etchant, the W^{6+} 4*f* peaks belonging to WO_{3-x} disappear, indicating that the WO_{3-x} nanowires are etched by KOH solution. The low-magnification TEM images also show the intact flat surface of the underlying WSe_2 layer after etching WO_{3-x} by KOH solution (Supplementary Fig. 14a-c). Besides, there are occasionally WO_{3-x} nanowires left after KOH etching (Supplementary Fig. 14d). Supplementary Fig. 15 displays the SAED patterns at various flakes of the underlying WSe_2 after immersing into KOH etchant. It can be seen that all of the SAED patterns exhibit hexagonal diffraction spots, illustrating the underlying WSe_2 layers reserving the pristine crystalline structure and the self-limited oxidation process in nature of the oxygen plasma treatment. And, the HRTEM images at various flakes also show the honeycomb lattice belonging to the underlying WSe_2 (Supplementary Fig. 16). Taken above, the oxygen plasma treatment of few-layer WSe_2 to form 1D/2D WO_{3-x}/WSe_2 heterostructures is a self-limited oxidation process, and the underlying WSe_2 can nearly remain the pristine crystalline structure.

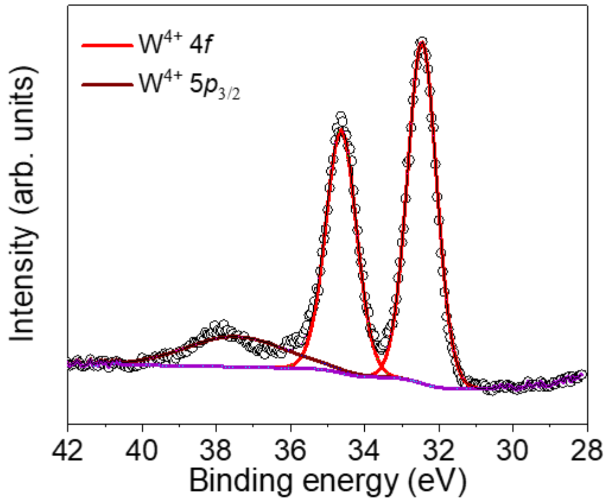

Supplementary Figure 13. XPS fine scan spectrum of the underlying WSe_2 after immersing 1D/2D WO_{3-x}/WSe_2 heterostructures into 1M KOH etchant. After immersing into 1M KOH etchant, there are no W^{6+} 4*f* core-level peaks in the W 4*f* fine scan spectrum of the underlying WSe_2, demonstrating that the WO_{3-x} nanowires are almost removed.

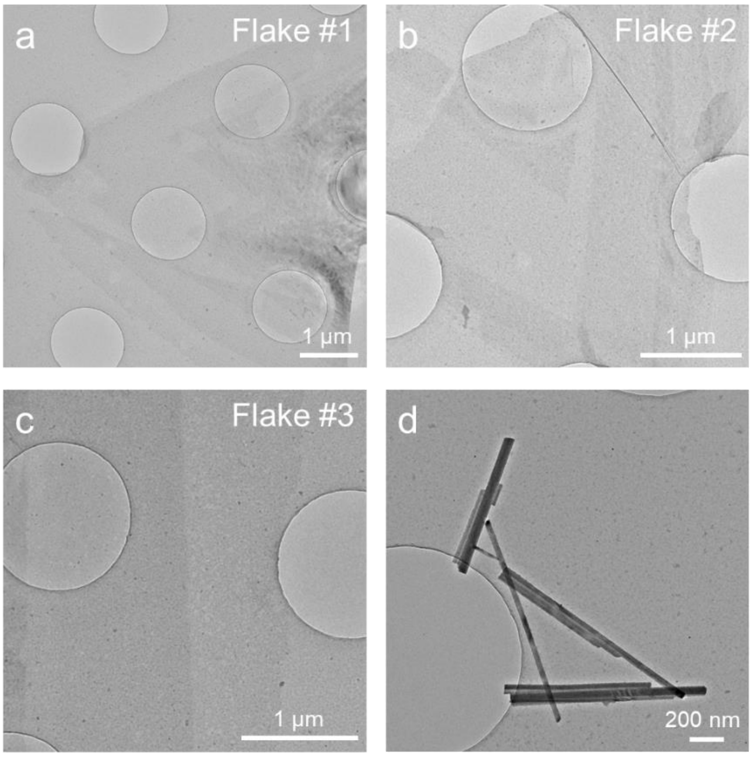

Supplementary Figure 14. (a-c) Typical TEM images of the underlying WSe_2 taken at different flakes after immersing 1D/2D WO_{3-x}/WSe_2 heterostructures into 1M KOH etchant to remove the surface WO_{3-x} nanowires. (d) Residual WO_{3-x} nanowires after KOH etching.

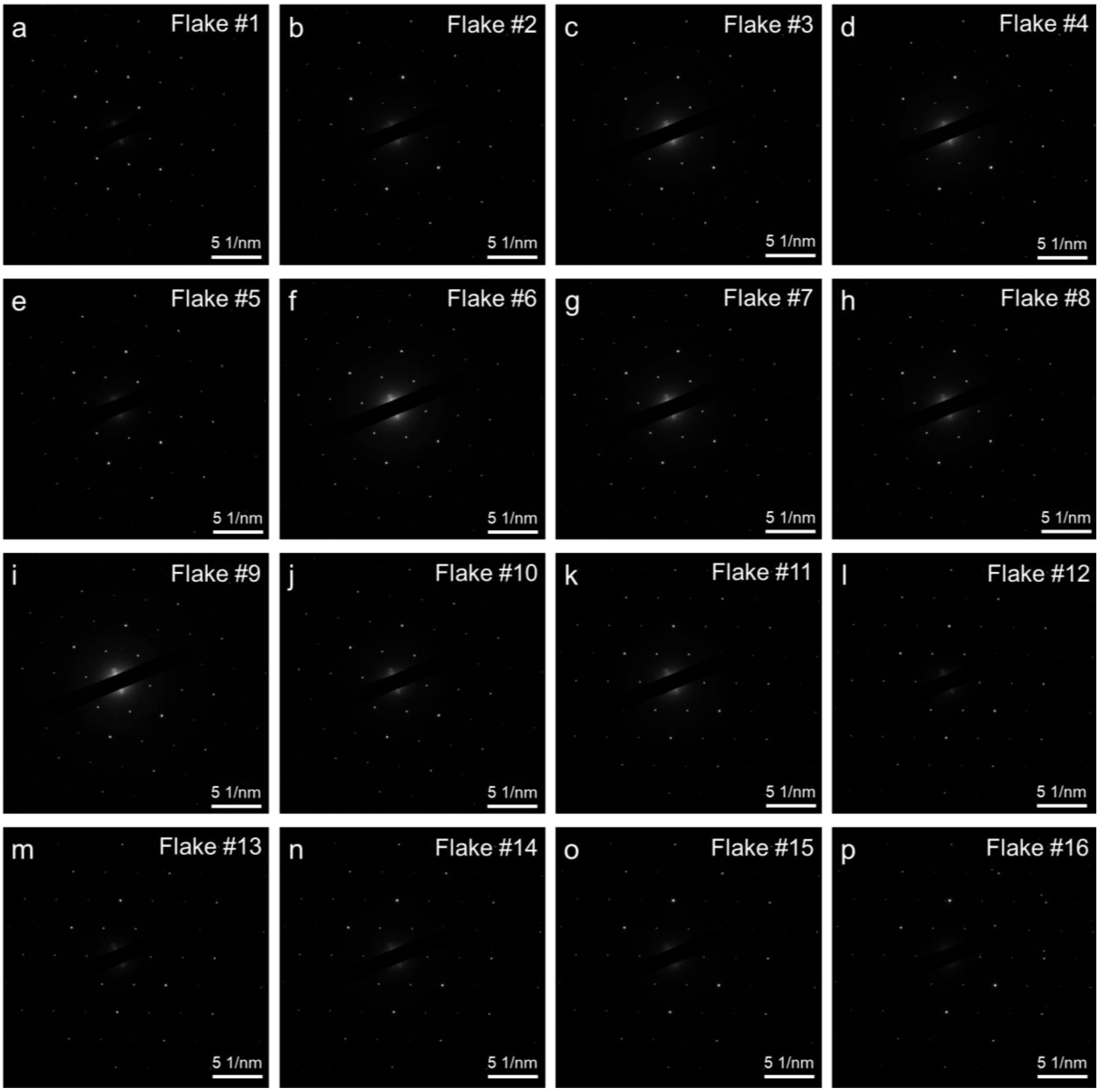

Supplementary Figure 15. (a-p) SAED patterns of the underlying WSe_2 taken at different flakes after immersing 1D/2D WO_{3-x}/WSe_2 heterostructures into 1M KOH etchant.

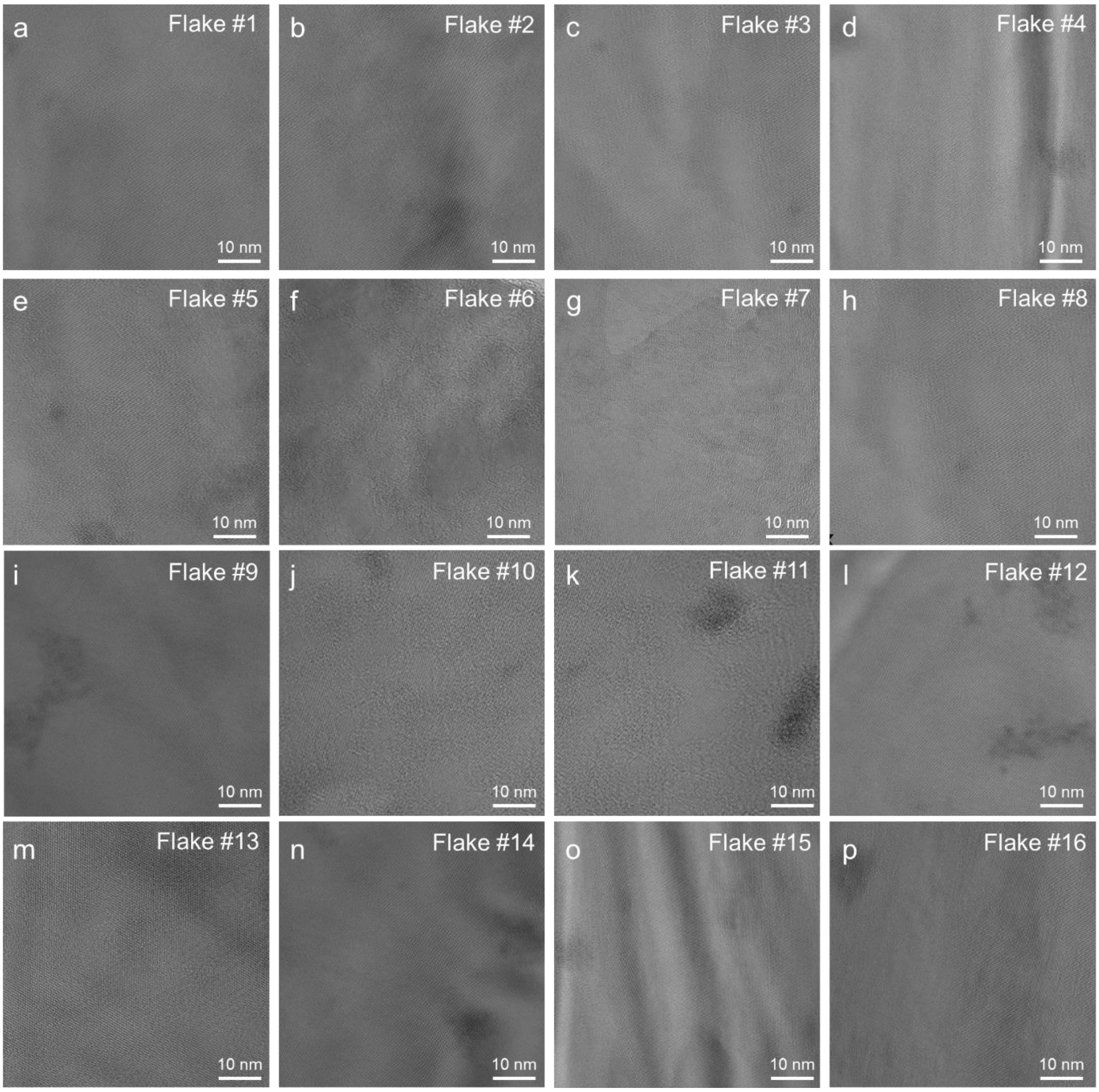

Supplementary Figure 16. (a-p) HRTEM images of the underlying WSe_2 taken at different flakes after immersing 1D/2D WO_{3-x}/WSe_2 heterostructures into 1M KOH etchant.

8. Strain maps at the interfaces of WO_{3-x} nanowires

In terms of the WO_{3-x} nanowires aligned along the three-fold symmetric directions of the WSe_2 (Supplementary Fig. 17a), there exist strain at the interfaces of WO_{3-x} nanowires due to the different crystallographic orientation. Thus, we identify and quantify the strain at the nanowire interfaces by using geometric phase analysis (GPA) method. It was carried out by the free FRWRtools plugin (see the link shown below) for Digital Micrograph software. Here, the strain field distributions were illustrated by the lattice fringes variations of the HRTEM image across the fields of view. To map the strain filed distribution, the unstrained area of nanowire II was chosen as the reference lattice with the direction x and y corresponding to [200] and [020] (Supplementary Fig. 17b), respectively. Thus, only the strain field distribution in the region related to nanowire II can be analyzed, which include I-II and III-II nanowire interfaces. Note that the GPA color maps on nanowire I and III based on the nanowire II unstrained area as reference lattice can not be used as the real evidence to reflect the strain field distribution. Supplementary Fig. 17c shows the ε_{xx} strain map, indicating the existence of x-direction normal strain at the interface between II and III nanowires. The ε_{yy} map in Supplementary Fig. 17d shows the y-direction normal strain, which is mainly distributed at the I-II and III-II nanowires interfaces. The ε_{xy} map in Supplementary Fig. 17e represents the shear strain, and the ε_{xy} is also mainly distributed at the interfaces of the I-II and III-II nanowires. According to the strain intensity profile (Supplementary Fig. 17f), the strain at the interfaces of WO_{3-x} nanowires is ~ 1%.

Link for FRWRtools plugin: www.physics.hu-berlin.de/en/sem/software/software_frwrtools

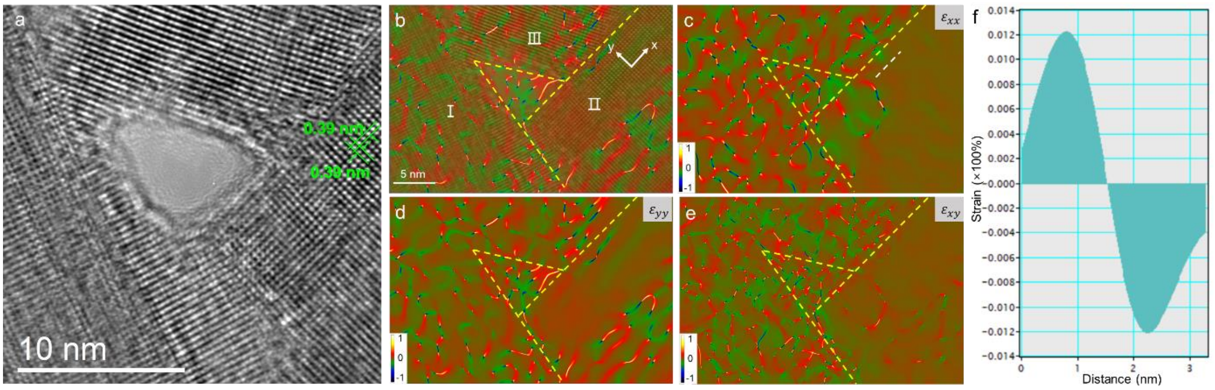

Supplementary Figure 17. Strain-field mapping at the interfaces of WO_{3-x} nanowires. (a) HRTEM image of three intersected WO_{3-x} nanowires. (b) A superimposed image and its ε_{yy} strain map. (c) ε_{xx}, (d) ε_{yy} and (e) ε_{xy} strain maps. (f) The corresponding strain intensity profile at the interfaces of WO_{3-x} nanowires along the whited dotted line in (c).

9. Initial transformation process from 2D WSe_2 flakes to 1D WO_{3-x} nanowires

The as-synthesized WSe_2 was treated by plasma with very short time (few seconds) to scrutinize the initial transformation process in Supplementary Fig. 18. Since the edges are much more chemically active than the inert basal plane in WSe_2, the edges of WSe_2 will be first converted to 1D WO_{3-x} nanowires even with a short plasma treatment duration of 2 s (Supplementary Fig. 18a). With increasing the plasma time to 10 s, the basal plane of WSe_2 can be gradually oxidized and converted to WO_{3-x} nanowires (Supplementary Fig. 18b-d).

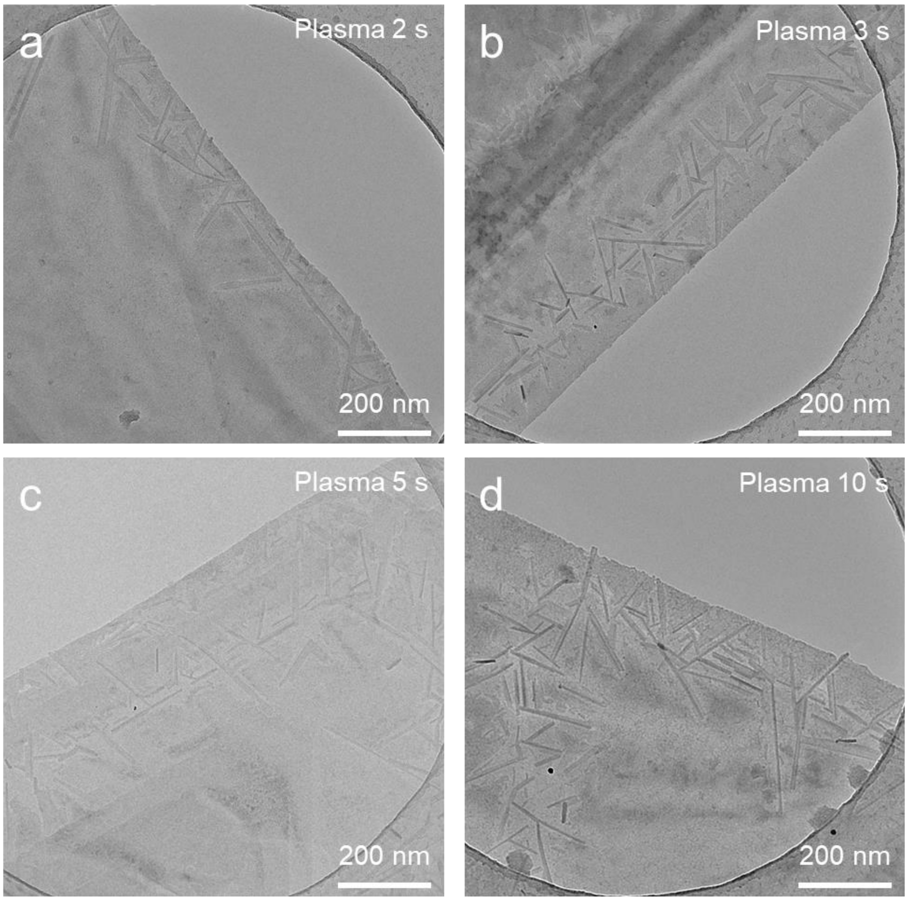

Supplementary Figure 18. Low-magnification TEM images of WSe_2 treated by plasma with different durations. (a) 2 s. (b) 3 s. (c) 5 s. (d) 10 s.

According to HAADF-STEM images of WSe_2 treated for 2-5 s, the top WSe_2 layer can be etched into notches with triangular-like and hexagonal-like shapes, which is consistent with the three-fold symmetry of pristine WSe_2 (Supplementary Fig. 19a-b). These etched WSe_2 will form separated small clusters with the etching of oxygen plasma (Supplementary Fig. 19c). Then, the structural reconstruction occurs under continuous plasma treatment, where these clusters can be crystallized into WO_{3-x} nanowires (Supplementary Fig. 19d). And, further increasing the oxygen plasma time to 10 s, it can be found that the WSe_2 layers around WO_{3-x} nanowires are usually one layer thinner than the parent materials (Supplementary Fig. 20), which proves that the constituent substance of these WO_{3-x} nanowires come from the oxidation of the etched WSe_2.

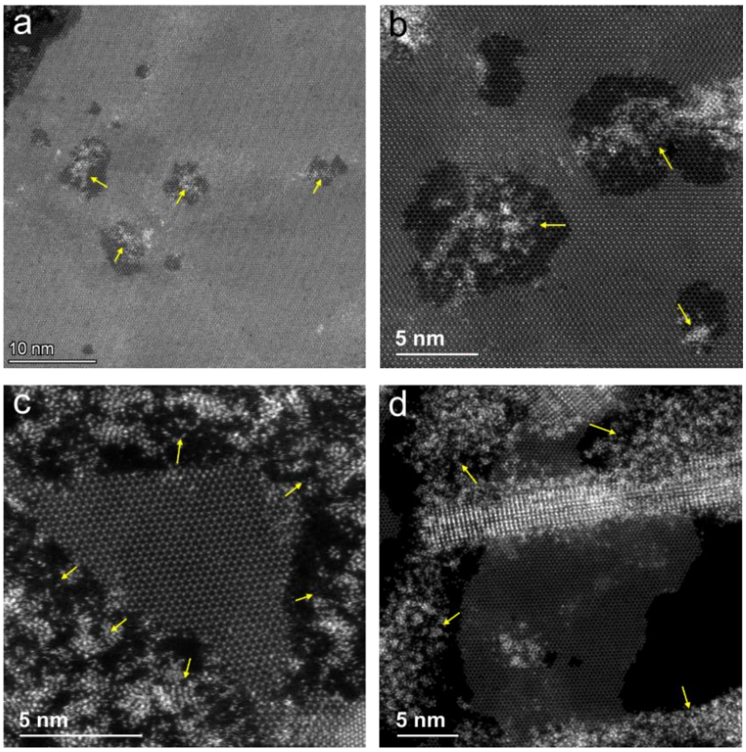

Supplementary Figure 19. HAADF-STEM images of WSe_2 treated by oxygen plasma for several seconds. (a-b) Etched regions of pristine WSe_2. (c-d) Formation of separated small clusters (c) and structural reconstruction from separated clusters to crystalline WO_{3-x} nanowires (d). The yellow arrows indicate the amorphous clusters coming from the etching of WSe_2.

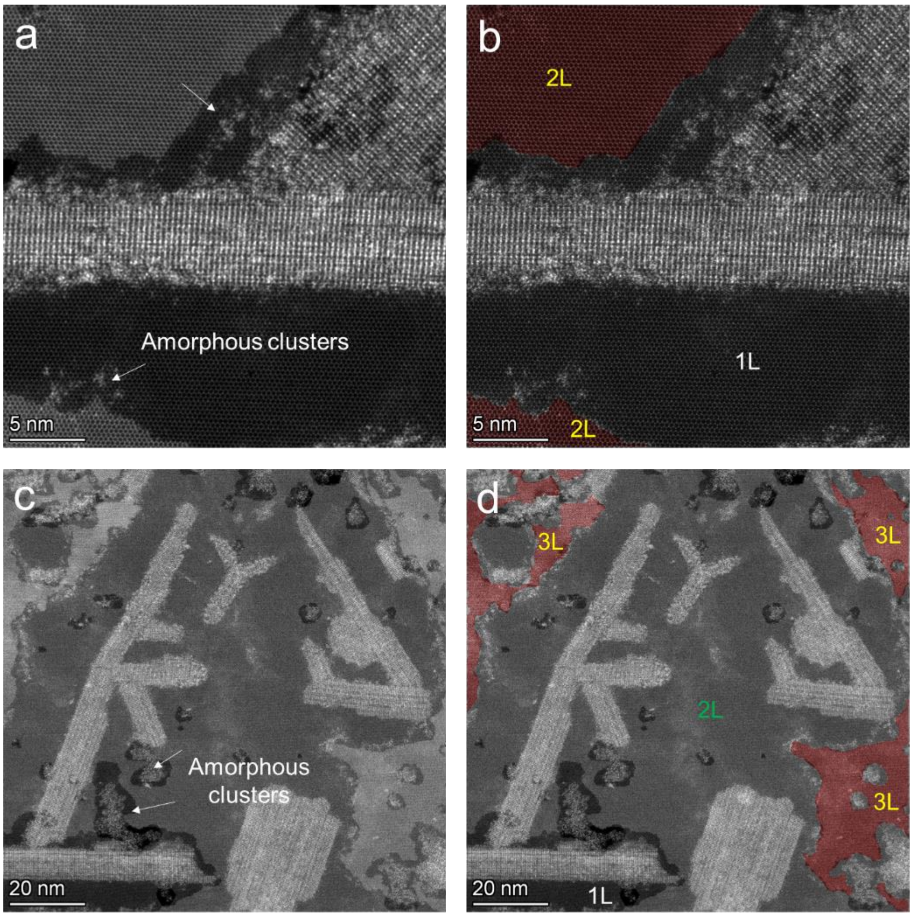

Supplementary Figure 20. HAADF-STEM images of WSe_2 treated by oxygen plasma for 10 s, where the structural reconstruction has occurred. The layer numbers of (a) and (c) were marked in (b) and (d), respectively.

10. Transformation mechanism based on density functional theory (DFT) calculations

Based on the preferential alignment of 1D WO_{3-x} nanowires and the three-fold symmetry of 2D WSe_2 lattice, the structural transformation mechanism was further verified by DFT calculations. Supplementary Fig. 21 illustrates the lattice reconstruction process from 2D WSe_2 flake to 1D WO_{3-x} nanowire induced by the oxygen plasma. Bombarded by ions with incident energy, the atoms in the lattice are sputtered, and thus form vacancies[4]. Thus, abundant Se vacancies are first formed after being treated by oxygen plasma (Supplementary Fig. 21b). Previous studies have also reported the employment of Ar and H_2 plasma to generate non-metal vacancies in TMDCs due to the lower formation energy of chalcogen vacancies than that of transition metal vacancies[5, 6]. DFT calculations reveal that the Se vacancies with linear arrangement are more energetically favorable than those with random distributions (Supplementary Fig. 22). The Se vacancy lines along the three directions with an angle of 120 ° are possible due to the three-fold symmetry of WSe_2 lattice (Supplementary Fig. 21c)[7]. Thus, the treated regions can be etched into the shapes with three-fold symmetry with the proceeding of oxygen plasma treatment. Subsequently, the reactive Se vacancies can be gradually substituted by oxygen atoms due to the decreased formation energy with the increasing of substitutional oxygen contents based on equation (1), as shown in Supplementary Figs. 23-24, corresponding to the oxygen substitution process of WSe_2 in Supplementary Fig. 21d, e. Therefore, it is energetically favorable that WSe_2 is converted to WO_{3-x} by oxygen plasma treatment. In terms of the different coordination environments of W atoms in WSe_2 and WO_{3-x} lattices, the atomic structures of WO_{3-x} with different lattice constructions are considered (Supplementary Fig. 25) and the corresponding formation energy of each construction was

calculated based on equation (2), as shown in Supplementary Fig. 26. With the increasing of W atom layers (N), the formation energy of WO_{3-x} decreases and converges to the formation energy of bulk WO_3 gradually. While for N⩾3, the formation of tetragonal WO_{3-x} is more energetically favored than that of hexagonal WO_2, with the Se atoms of WSe_2 directly substituted by O atoms. Therefore, the lattice transformation of WO_{3-x} from three-fold symmetric lattice to four-fold symmetric lattice is feasible, including the breaking of chemical bonds and changes of bond angle and bond length, leading to the formation of oriented 1D nanowires preferentially along the three-fold symmetric directions of WSe_2 (Supplementary Fig. 21f). It is speculated that the laterally oriented 1D WO_{3-x} nanowire patterns can be converted from 2D WSe_2 flake through the generation and linear arrangement of Se vacancies, the oxygen substitution at Se vacancy sites and the lattice reconstruction driven by oxygen plasma treatment.

The formation energy of monolayer WSe_2 with the Se atoms substituted by oxygen with different percentages was calculated using the following formula:

$$E_f = [E(W_{78}Se_{176-x}O_x) - E(W_{78}Se_{176}) + x\mu_{Se} - x\mu_O] / x \quad (1)$$

where $E(W_{78}Se_{176-x}O_x)$ and $E(W_{78}Se_{176})$ are the total energies of $W_{78}Se_{176-x}O_x$ and $W_{78}Se_{176}$ with triangular structures, respectively. x is the number of substituted O atoms. μ_{Se} and μ_O are the chemical potentials for Se and O species, respectively.

The formation energy of WO_{3-x} with different lattice constructions was calculated using the following formula:

$$E_f = [E(W_xO_y) - E(W_x) - E(O_y)] / (x + y) \quad (2)$$

where $E(W_xO_y)$ is the total energy of the W_xO_y compound, and $E(W_x)$ and $E(O_y)$ are the energies

per atom for W and O in their stable phase, respectively.

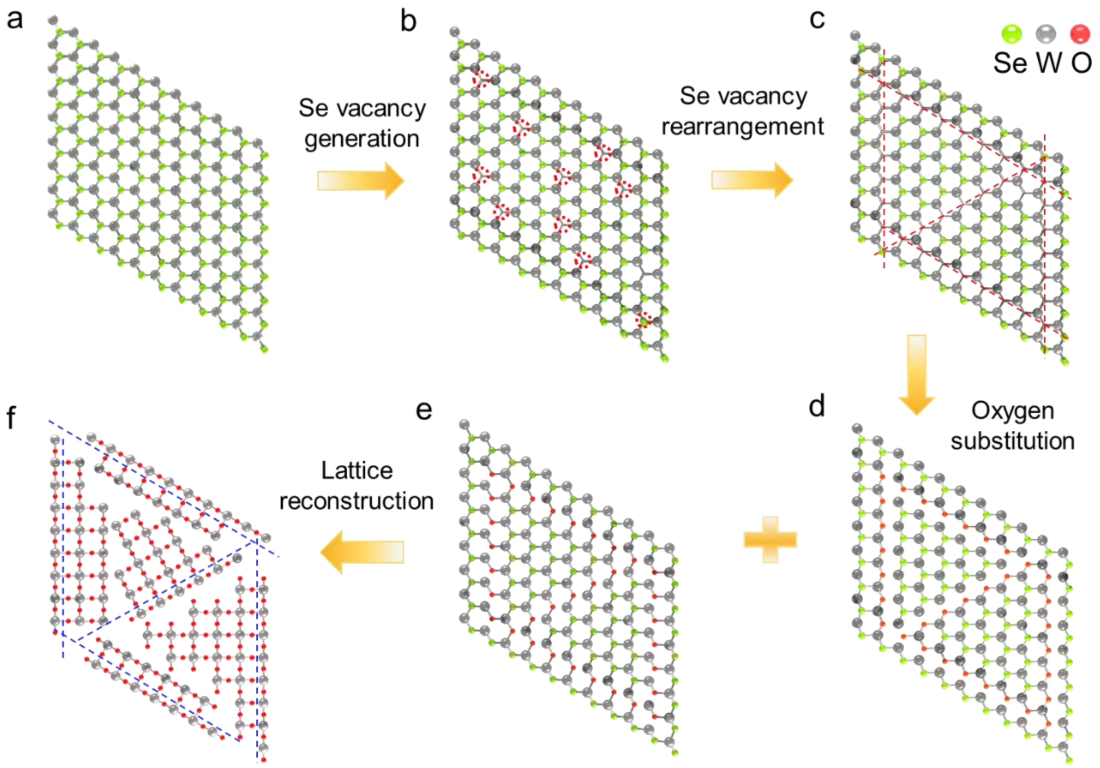

Supplementary Figure 21. Schematic illustration of the lattice reconstruction process from 2D WSe_2 flakes to 1D WO_{3-x} nanowires. Red dotted circles represent the Se vacancy sites. (a) Atomic structure of pristine WSe_2. (b) Generation of Se vacancies in WSe_2. (c) Linear arrangement of Se vacancies in WSe_2. (d-e) Oxygen substitution at the Se vacancies. (f) WO_{3-x} nanowires formation through the lattice reconstruction.

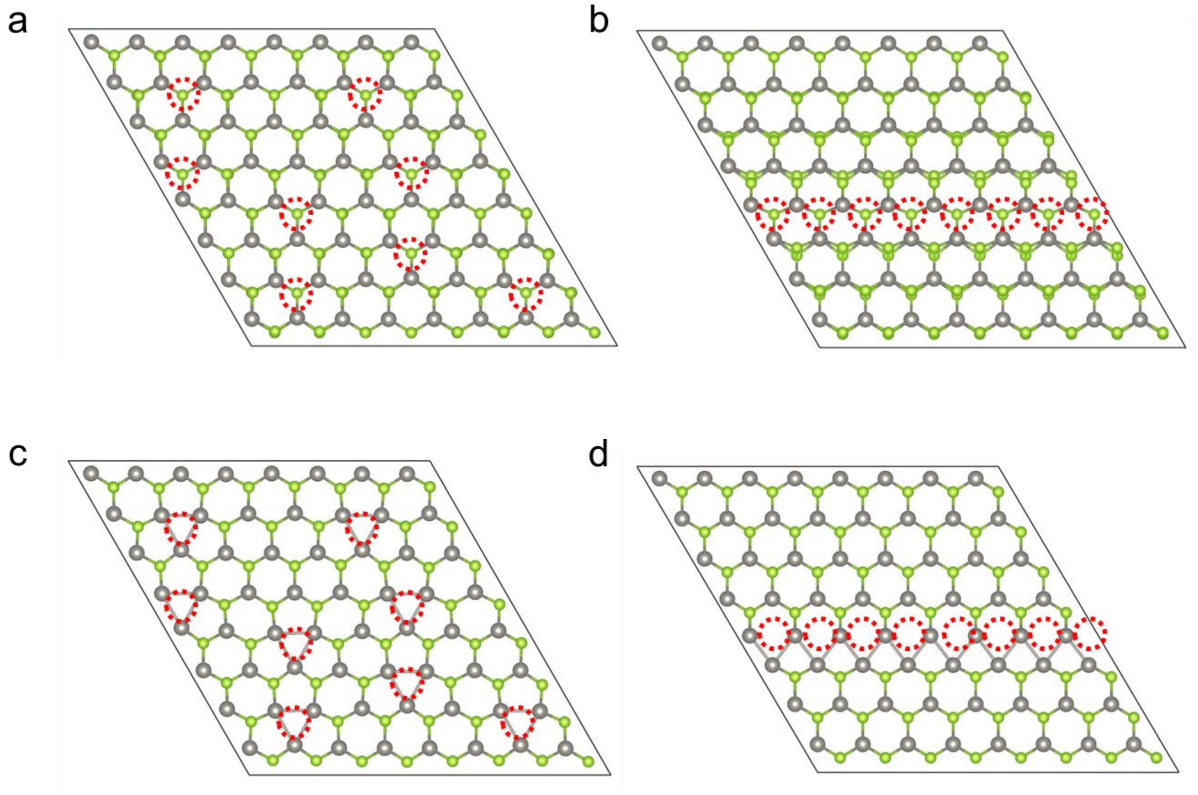

Supplementary Figure 22. Atomic structures of monolayer WSe_2 with (a-b) 6.25% (upper) and (c-d) 12.5% (lower) Se vacancies. Red dotted circles represent the Se vacancy sites. (a, c) Se vacancies with random distribution. (b, d) Se vacancies with linear arrangement. Density functional theory (DFT) calculations demonstrate that the Se vacancies with linear arrangement are more energetically favored by 0.51 eV and 0.04 eV per Se vacancy for 6.25% and 12.5% than those with random distributions, respectively.

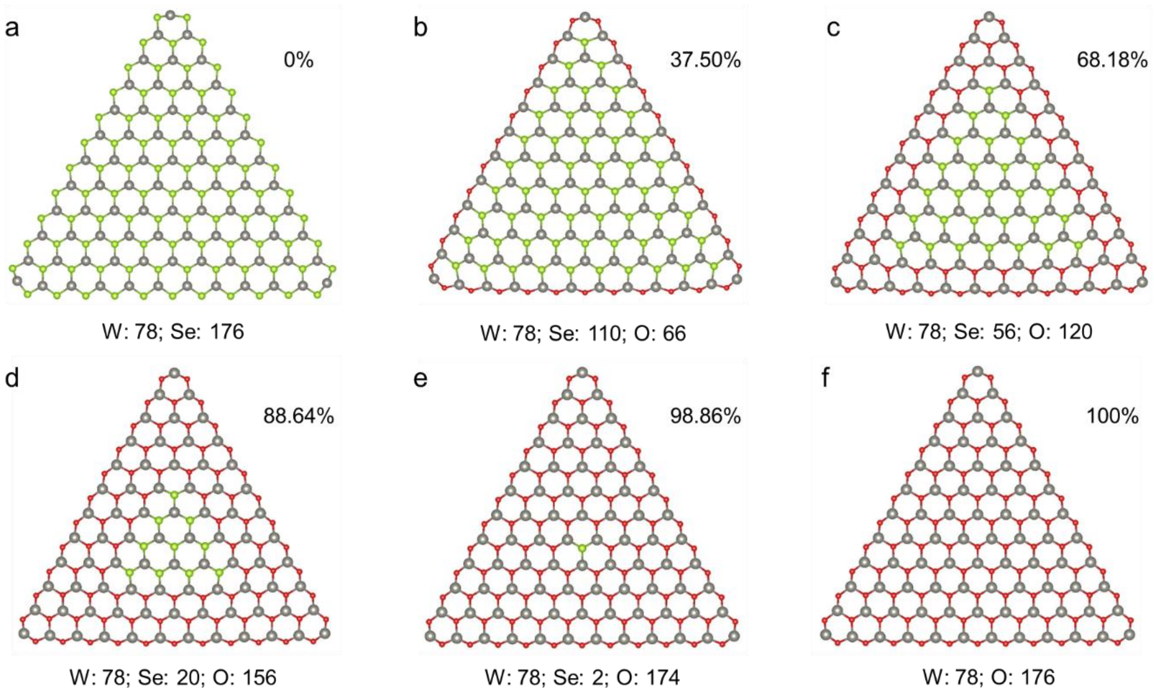

Supplementary Figure 23. Atomic structures of monolayer WSe_2 with the Se atoms gradually substituted by oxygen atoms with different percentages. (a) 0%, (b) 37.50%, (c) 68.18%, (d) 88.64%, (e) 98.86%, (f) 100%.

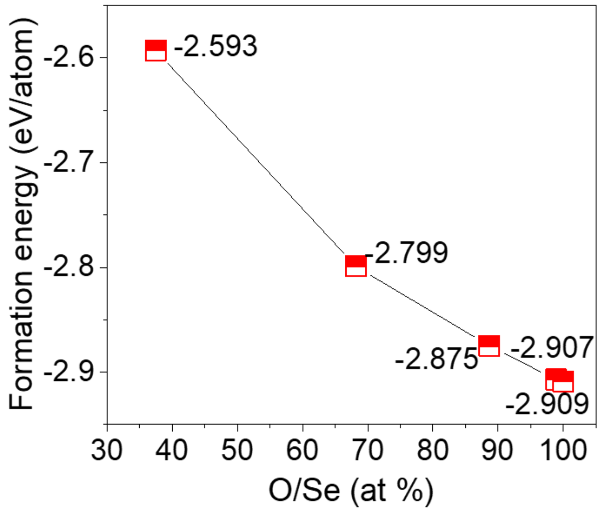

Supplementary Figure 24. Formation energy of WSe_2 with different Se vacancy percentages substituted by oxygen atoms.

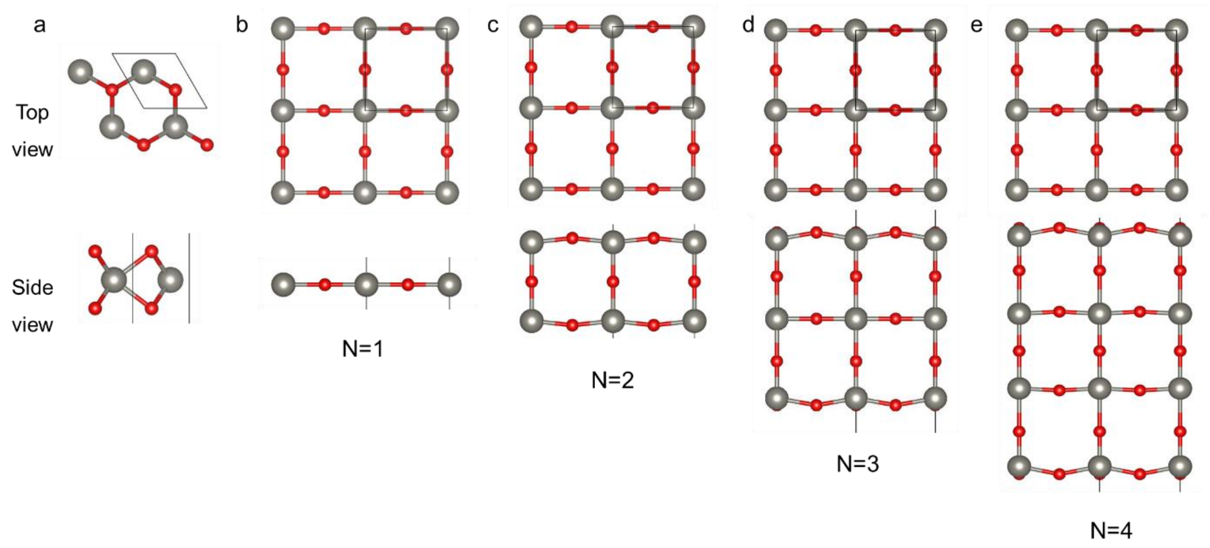

Supplementary Figure 25. Atomic structures of WO_{3-x} with different lattice constructions. (a) WO_{3-x} with hexagonal lattice. (b-e) WO_{3-x} with tetragonal lattice with different W atom layers (N).

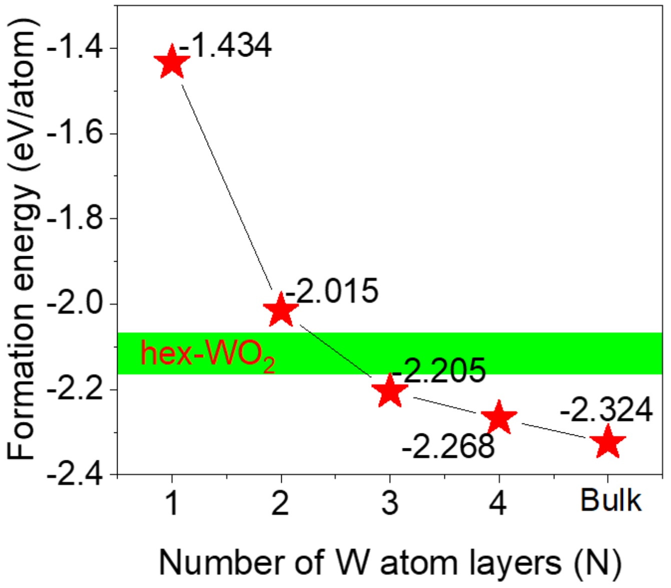

Supplementary Figure 26. Formation energy of WO_{3-x} with different number of W atom layers (N). Green region represents the energy window of hexagonal WO_2 (hex-WO_2) from monolayer to bulk.

11. Molecular sensing performance of samples

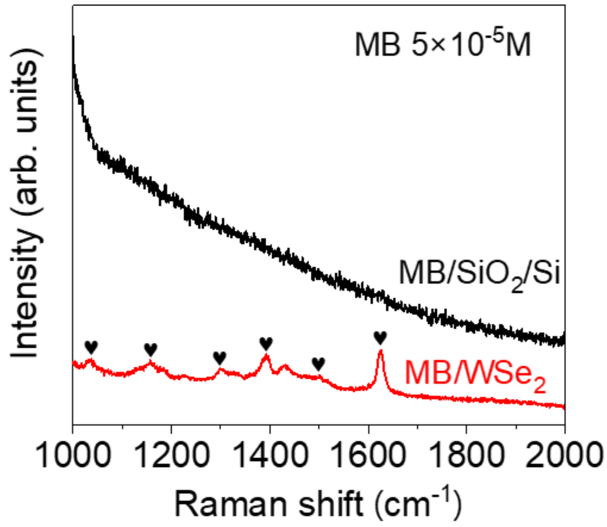

Supplementary Figure 27. Raman spectra of MB molecules on bare SiO_2/Si substrate and pristine WSe_2. Due to the strong fluorescence background of MB molecules, Raman fingerprints of MB on bare SiO_2/Si are hardly detected. In contrast, the MB fingerprints can be clearly distinguished on pristine WSe_2 with greatly quenched fluorescence background, indicating the enhanced Raman scattering effect of pristine WSe_2.

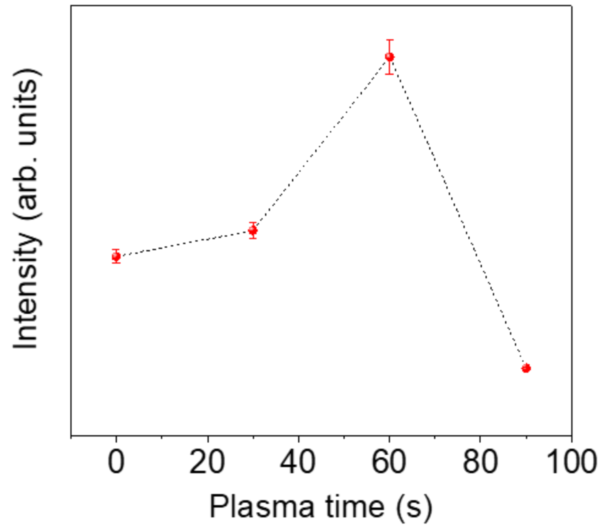

Supplementary Figure 28. Raman intensities of MB at 1620 cm^{-1} on WSe_2 with different plasma treatment durations. Error bars: standard deviation (SD).

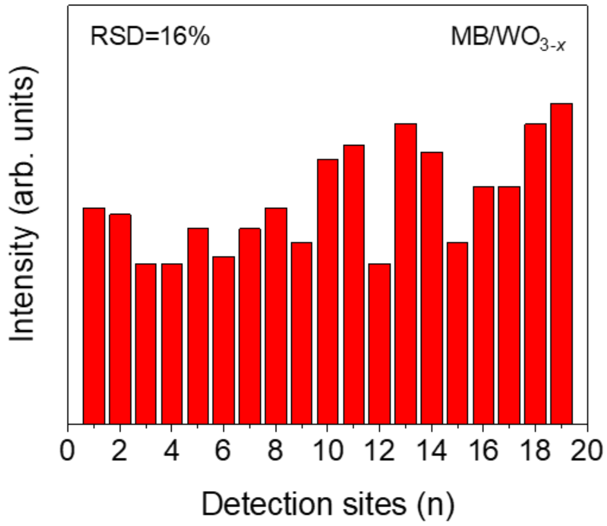

Supplementary Figure 29. Raman intensity distribution of MB molecules peaks at 1621 cm^{-1} on 1D/2D WO_{3-x}/WSe_2 heterostructures. RSD: Relative standard deviation.

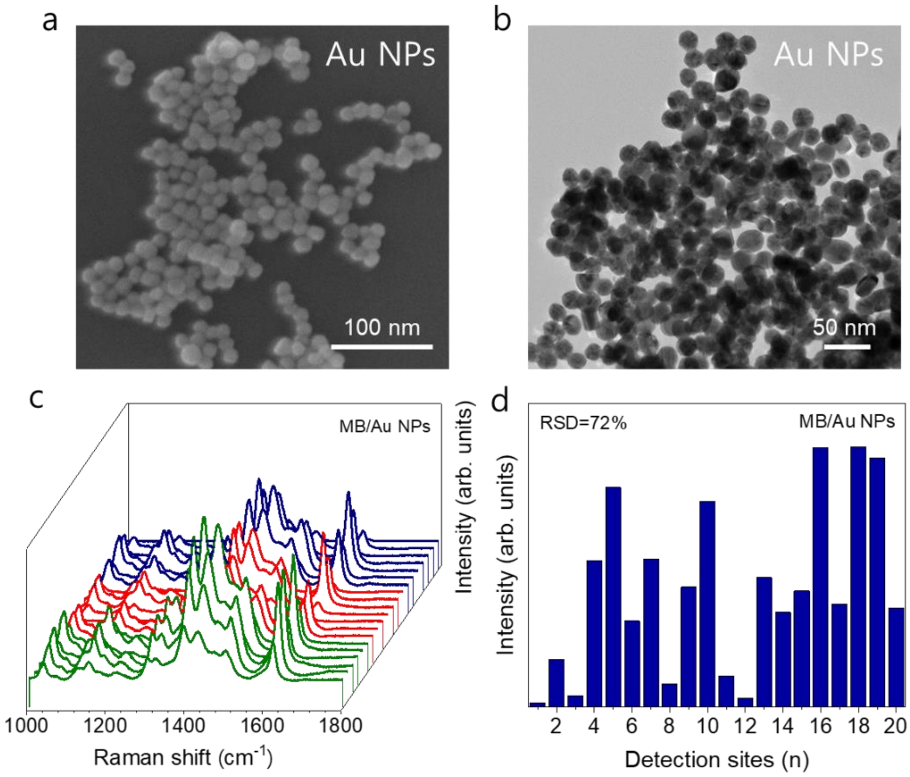

Supplementary Figure 30. (a) SEM and (b) TEM images of Au nanoparticles (Au NPs). (c) Raman spectra of MB molecules with the concentration of 5×10^{-5} M on Au NPs randomly selected at 20 points. (d) Raman intensity distribution of MB molecules peaks at 1621 cm^{-1} on Au NPs.

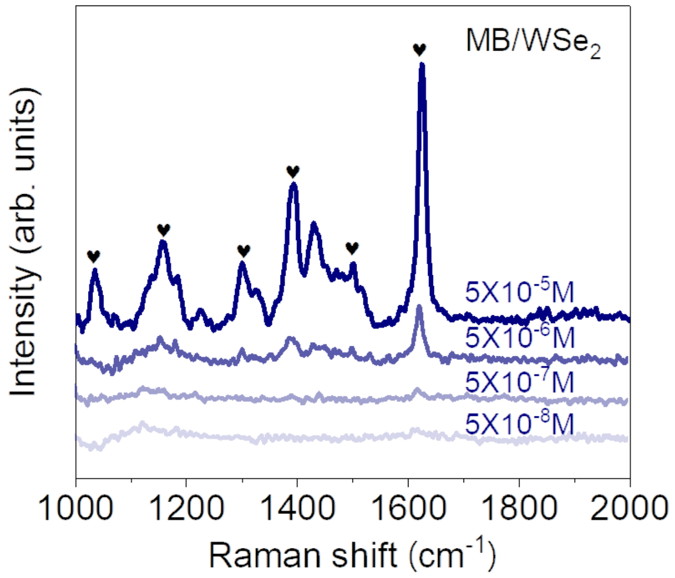

Supplementary Figure 31. Raman spectra of MB molecules on pristine WSe_2 with different concentrations.

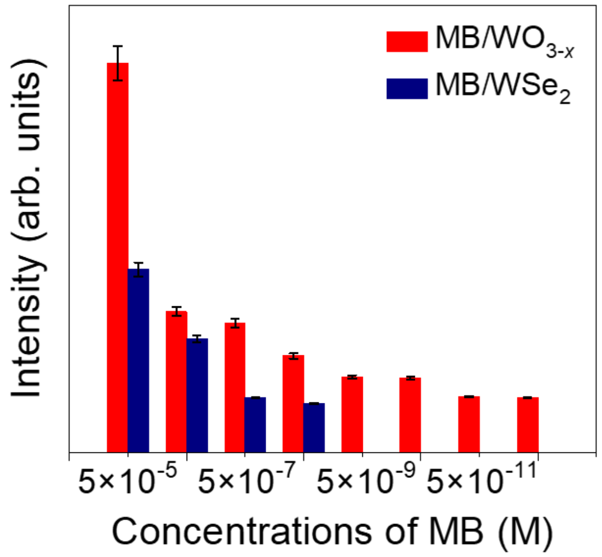

Supplementary Figure 32. Raman intensity evolution of MB with different concentrations on pristine WSe_2 and $WO_{3\text{-}x}$ at 1620 cm^{-1}. Error bars: SD.

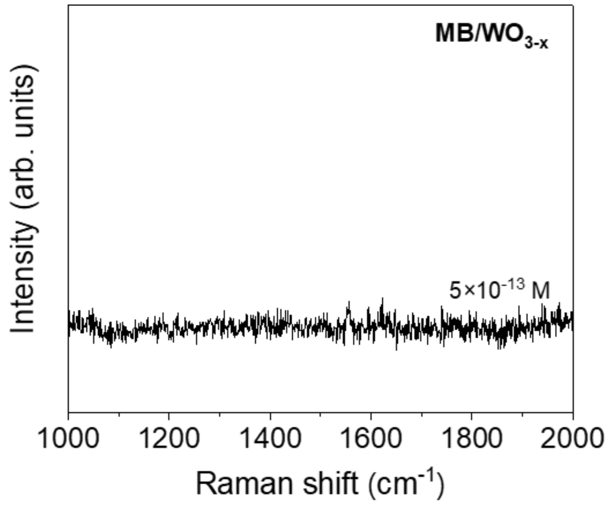

Supplementary Figure 33. Raman spectrum of MB molecules with the concentration of 5×10^{-13} M on 1D WO_{3-x} with the integration time of 40 s.

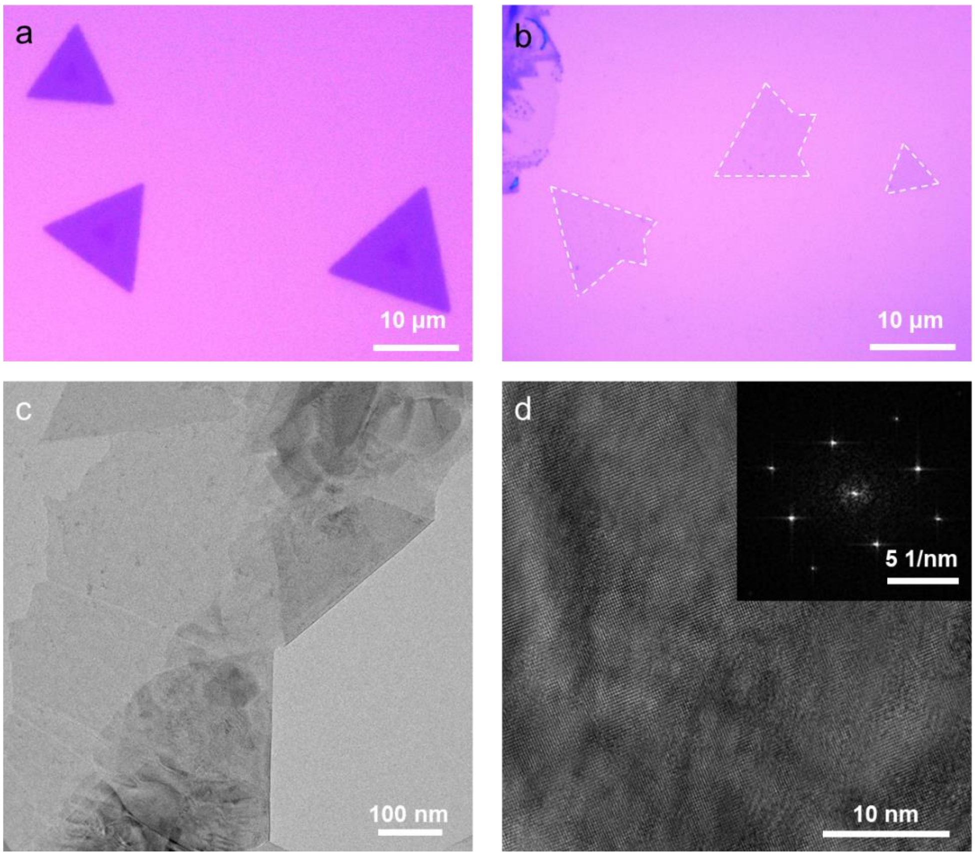

Supplementary Figure 34. Characterizations of WO_{3-x} nanoflakes (WO_{3-x}-NF). (a-b) Optical images of WSe_2 before (a) and after (b) annealing at 300 °C for ~ 6 min. (c) Low-magnification TEM image of WO_{3-x}-NF. (d) HRTEM image of WO_{3-x}-NF. Inset corresponds the FFT pattern.

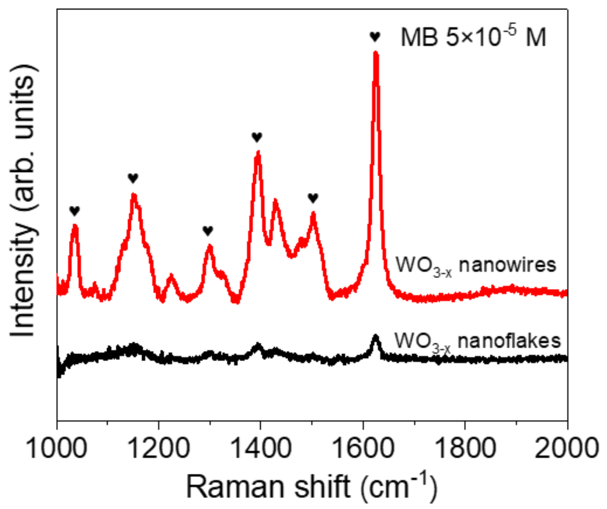

Supplementary Figure 35. Raman spectra of MB on $WO_{3\text{-}x}$ nanowires and $WO_{3\text{-}x}$ nanoflakes.

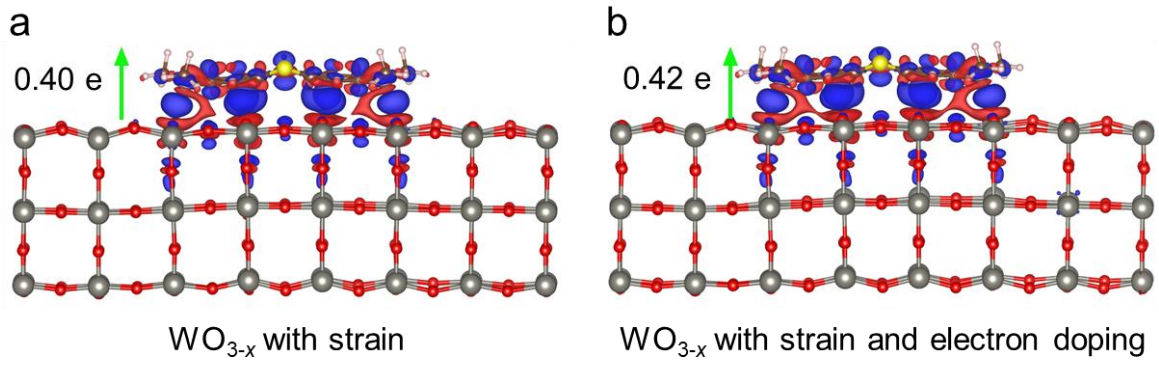

Supplementary Figure 36. Charge density differences of MB molecules on the $WO_{3\text{-}x}$ with (a) strain and (b) both strain and electron doping. The isosurface values is 0.0003 e/A^3.

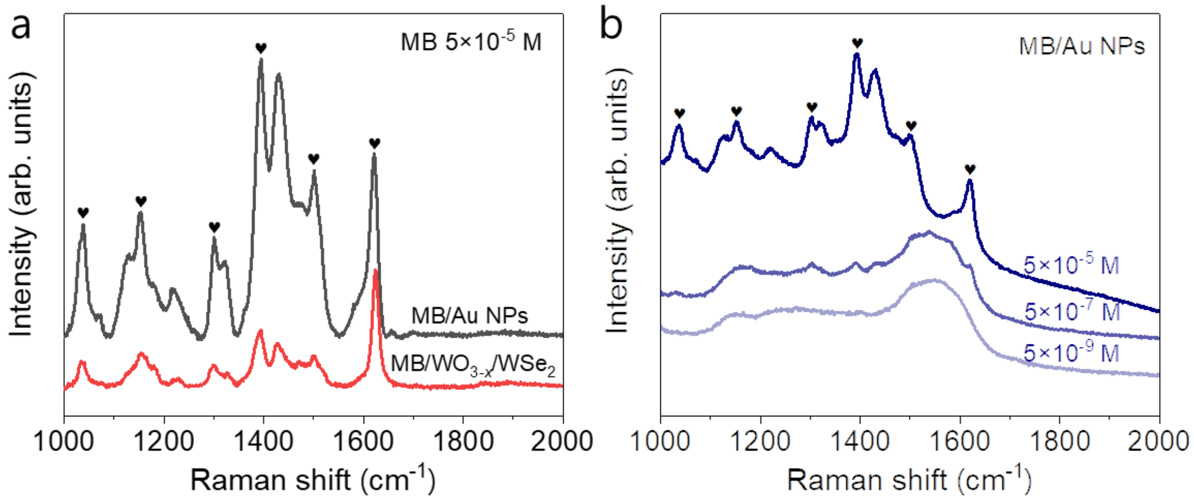

Supplementary Figure 37. (a) Raman spectra of MB molecules with the concentration of 5×10^{-5} M on Au NPs and WO_{3-x}/WSe_2. (b) Raman spectra of MB molecules with different concentrations on Au NPs.

The enhancement factor (EF) can be calculated according to the following equation[8, 9]:

$$EF=(I_{SERS}/N_{SERS})/(I_{bulk}/N_{bulk}) \quad (3)$$

$$N_{SERS}=cvN_{A}A_{1}/A_{sub} \quad (4)$$

$$N_{bulk}=\rho hN_{A}A_{1}/M \quad (5)$$

WWhere I_{SERS} and I_{bulk} are the Raman intensities of probe molecules on SERS substrate and bulk molecules on bare substrate, respectively. N_{SERS} and N_{bulk} refer to the amounts of probe molecules in the SERS test and in the bulk Raman test, respectively. V and c are the volume and concentration of probe molecules, respectively. M and ρ are the molar mass and density of probe molecules (319.86 g mol^{-1}, 1.757 g cm^{-3} for MB), respectively. A_1 is the illumination area of Raman laser. The layer penetration depth (h) can be calculated by the equation of $h=2\lambda/NA^2$, in which NA is the numerical aperture of 0.5. The Raman intensity of MB on WO_{3-x}/WSe_2 with 40 s integration time is 393 counts and for bulk MB with 1 s integration time is 1374 counts (Supplementary Fig. 38). The number of bulk MB molecules can be calculated using the density of bullk MB due to the high concentration of MB solution (5×10^{-2} M). Thus, the EF of MB on WO_{3-x}/WSe_2 can be calculated as:

$$EF=\frac{I_{SERS}}{I_{bulk}}\times\frac{\rho hA_{sub}}{cVM}=\frac{393/40}{1374/1}\times\frac{1.757\times5.064\times10^{-4}\times0.25}{5\times10^{-18}\times2\times10^{-3}\times319.86}\approx5.0\times10^{11}$$

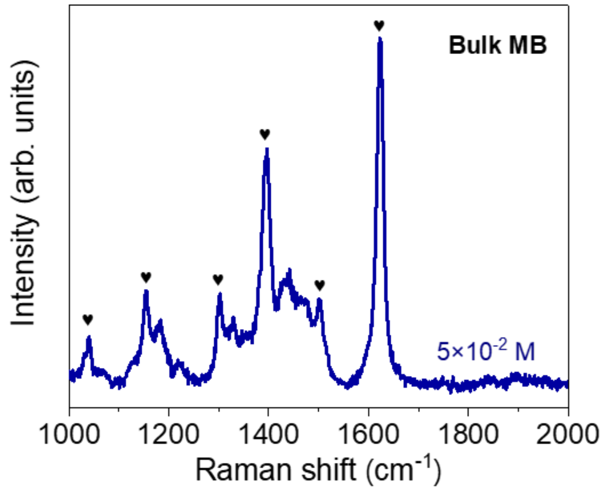

Supplementary Figure 38. Raman spectra of bulk MB with concentration of 5×10^{-2} M on bare SiO_2/Si substrate.

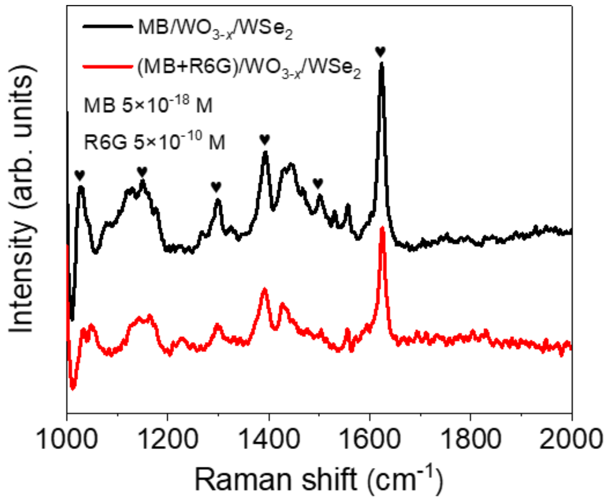

Supplementary Figure 39. Raman spectra of individual MB and MB in the mixed solution with R6G on 1D/2D $WO_{3\text{-}x}$/WSe_2 heterostructures. "♥" represents the characteristic Raman fingerprints of MB molecules.

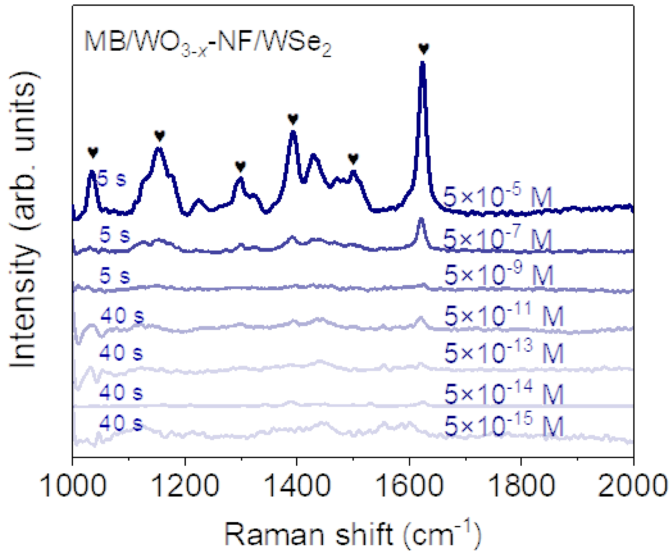

Supplementary Figure 40. Raman spectra of MB molecules with different concentrations on 2D/2D WO_{3-x}-NF/WSe_2 heterostructures. With an integration time of 5 s, the Raman intensity of MB molecules was very weak when the concentration reached 5×10^{-9} M. By increasing the integration time to 40 s, the LOD of WO_{3-x} NF/WSe_2 can be decreased to 5×10^{-14} M. Further decreasing the concentration of MB to 5×10^{-15} M, the Raman fingerprints of MB on WO_{3-x}-NF/WSe_2 cannot be detected.

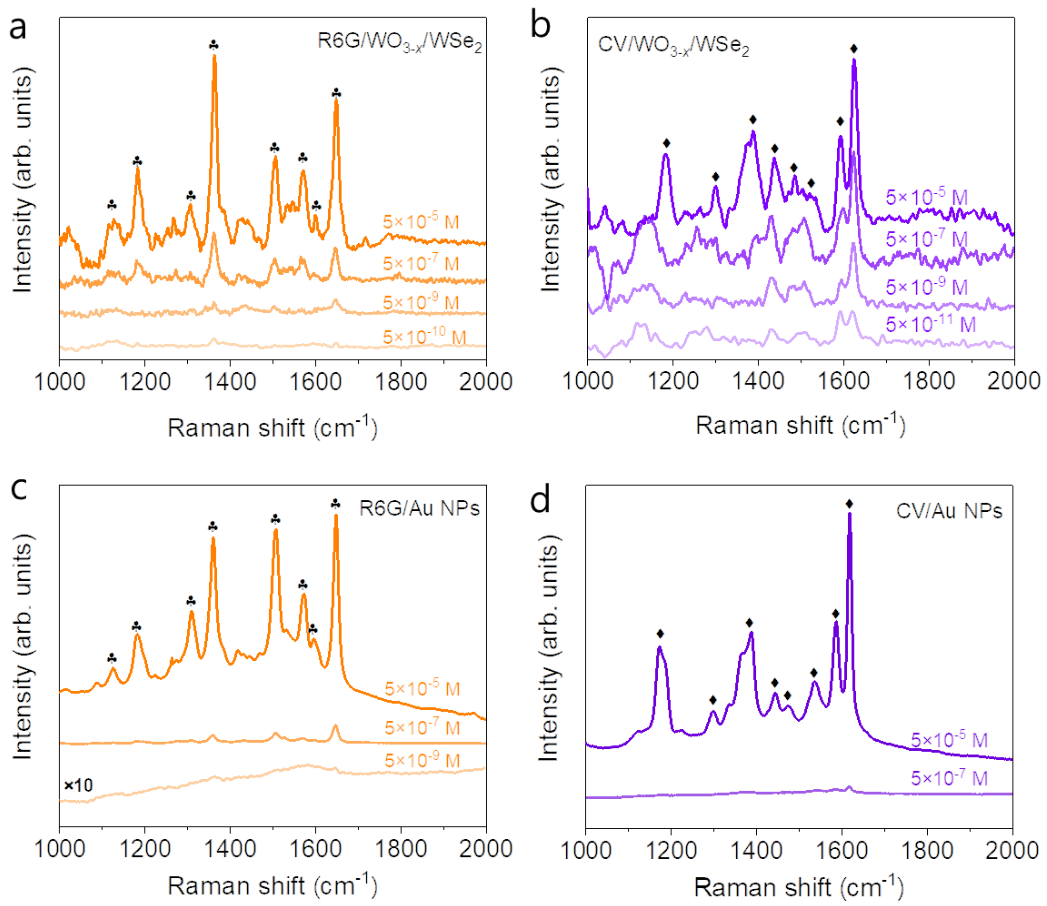

Supplementary Figure 41. Raman spectra of 1D/2D WO_{3-x}/WSe_2 heterostructures and Au NPs for probing different molecules. (a) Rhodamine 6G (R6G) and (b) Crystal Violet (CV) on WO_{3-x}/WSe_2 heterostructures. (c) R6G, and (d) CV on Au NPs. The distinct Raman fingerprints of R6G and CV molecules (marked by "♣" and "◆", respectively) demonstrate the universal sensing capability of WO_{3-x}/WSe_2 heterostructures for detecting various dye molecules.

When excited by 633 nm Raman laser, only the Raman fingerprints of MB molecules in the mixed solution can be observed on WO_{3-x}/WSe_2 substrate. When using 532 nm Raman laser, both the Raman fingerprints of MB and R6G molecules can be excited, achieving the excellent selectivity detection, which benefits the practical applications of WO_{3-x}/WSe_2 heterostructure in the future. The selective detection capability can be explained using the molecular transition resonance, namely, the matched molecular wavelength with the excited Raman laser wavelength ($\lambda_{mol} \approx \lambda_L$), which can contribute to increasing the Raman scattering cross-section[10]. Thus, this Raman laser dependent behavior can be related to the affinity levels of different molecules. The highest occupied molecular orbital (HOMO) and the lowest unoccupied molecular orbital (LUMO) levels of MB (R6G) are located at -6.26 (-5.70) and -4.55 (-3.40) eV, respectively. So, the energy difference of MB (R6G) between HOMO and LUMO is 1.71 (2.30) eV. Thus, 633 nm Raman laser can only excite the fingerprints of MB molecules, while 532 nm Raman laser can excite both the fingerprints of MB and R6G molecules.

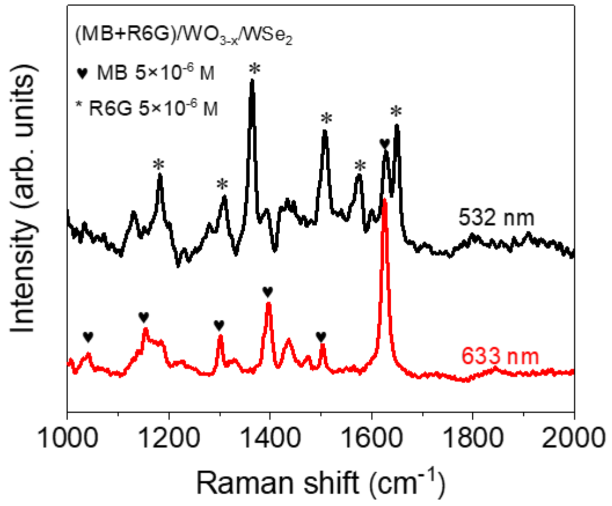

Supplementary Figure 42. Raman spectra of MB and R6G in the mixed solution collected on 1D/2D WO_{3-x}/WSe_2 heterostructure excited by 633 and 532 nm Raman laser.

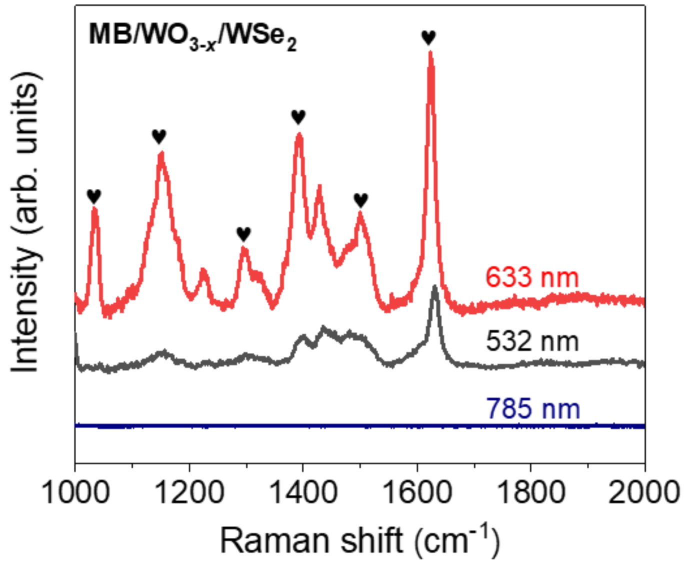

Supplementary Figure 43. Raman spectra of MB molecules on WO_{3-x}/WSe_2 heterostructures excited by different laser lines.

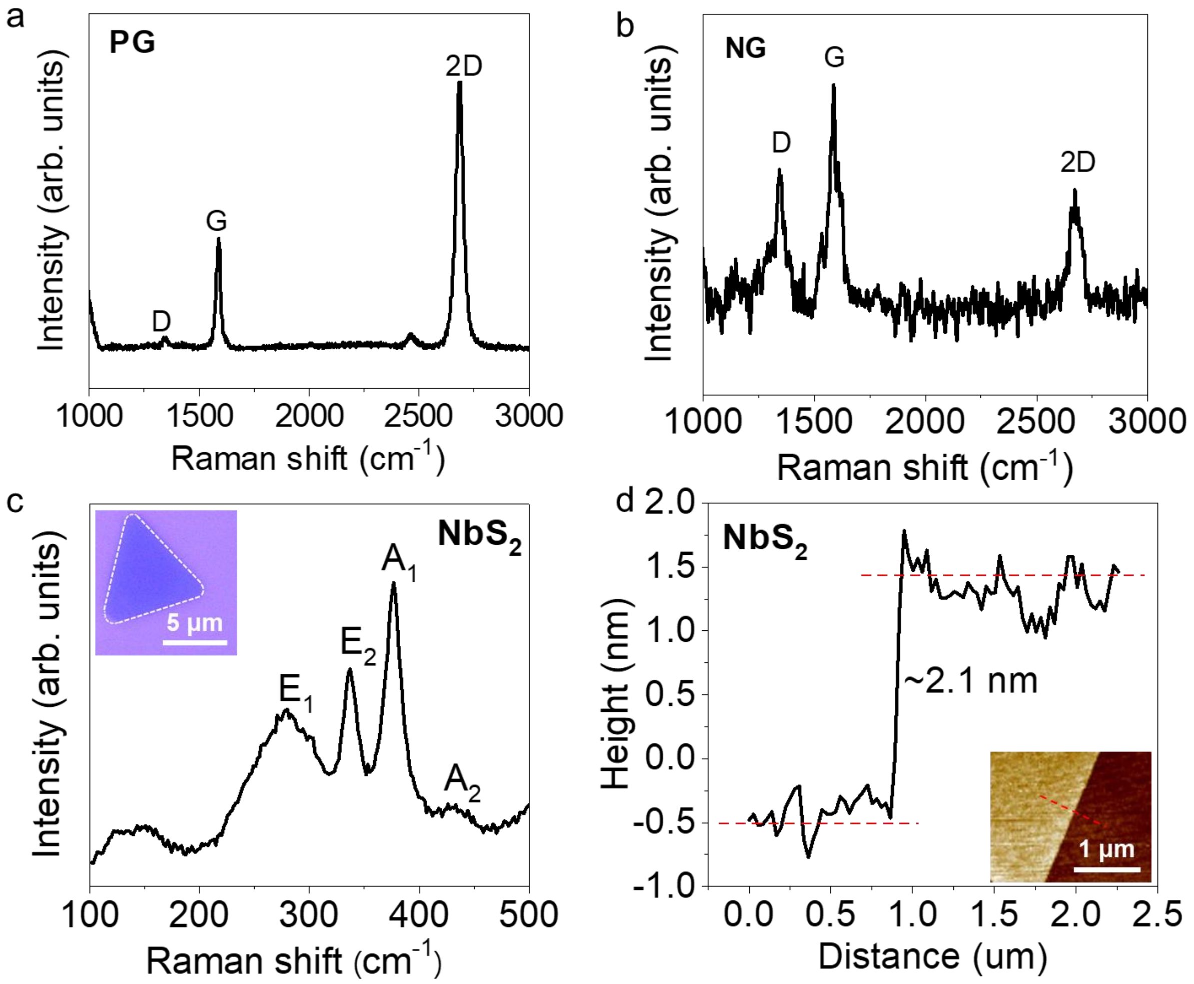

Supplementary Figure 44. Morphology and structure characterizations of other two-dimensional materials grown by AP-CVD. (a-b) Raman spectra of (a) pristine graphene (PG) and (b) nitrogen-doped graphene (NG). (c) Raman spectrum and (d) AFM height profile of few-layer NbS_2. Inset of (c) is the corresponding optical image. Inset of (d) is the AFM topological morphology. The E_1, E_2, A_1 and A_2 peaks are coincided with previous report[11]. The thickness of as-grown NbS_2 is ~2.1 nm according to the height profile, indicating that NbS_2 is 2-3 layers.

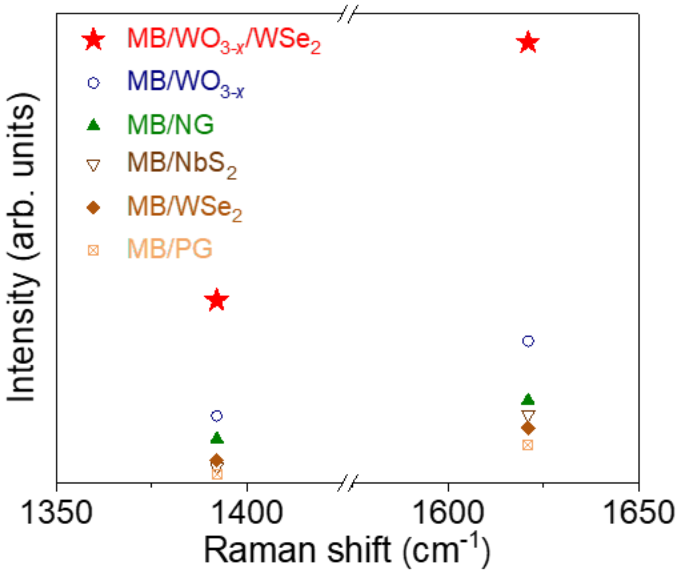

Supplementary Figure 45. Raman intensity comparison of MB molecules on different substrate materials.

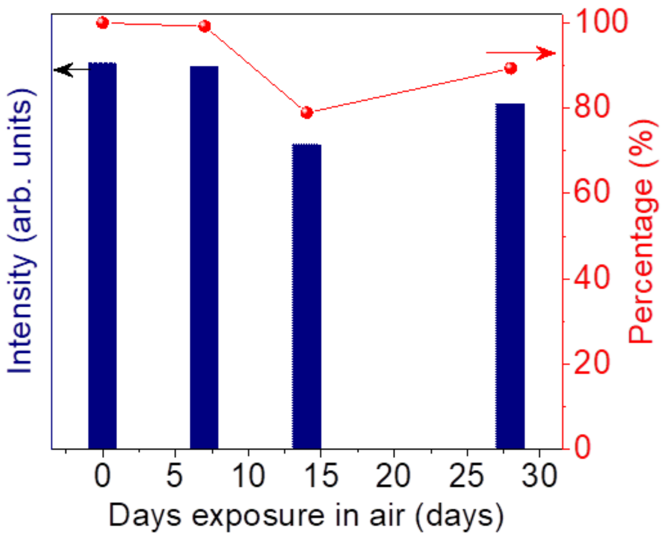

Supplementary Figure 46. Raman intensities and retention of MB on WO_{3-x}/WSe_2 heterostructures at 1620 cm^{-1} after being exposed in ambient conditions for different durations.

12. Ultrafast pump-probe transient spectroscopy analysis

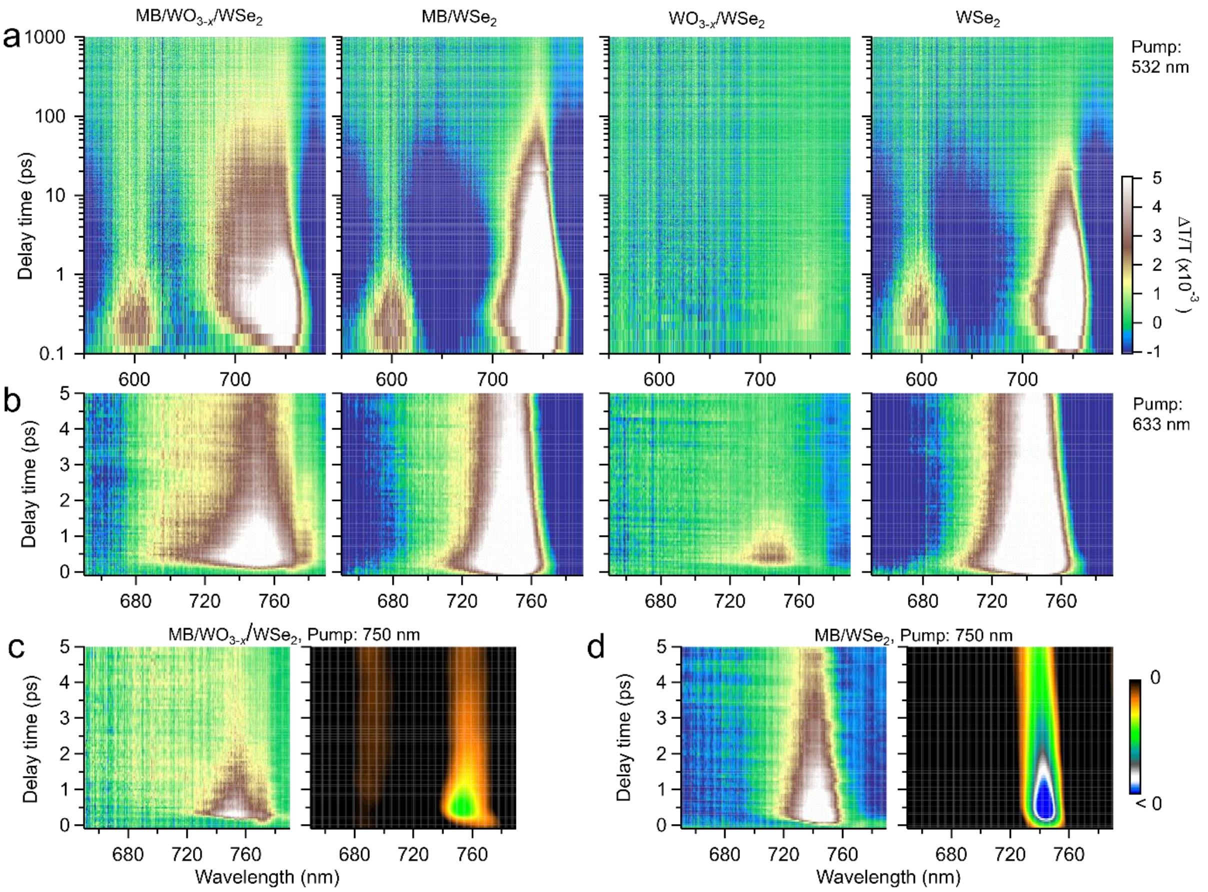

Supplementary Figure 47. Ultrafast transient transmission images of MB/WO_{3-x}/WSe_2, MB/WSe_2, WO_{3-x}/WSe_2 and WSe_2, by using different pump lights: 532 nm (a), 633 nm (b), 750 nm (c and d). The right panels in (c) and (d) are second derivative images with respect to the wavelength.

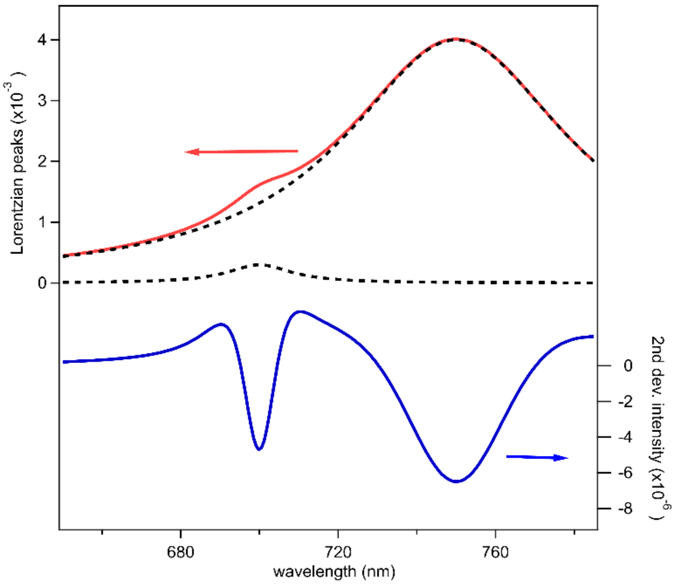

Supplementary Figure 48. Raw Lorentzian and second derivative curves. The second derivative curve effectively highlights both Lorentzian peaks (one weak and one strong), in terms of sharper negative dips.

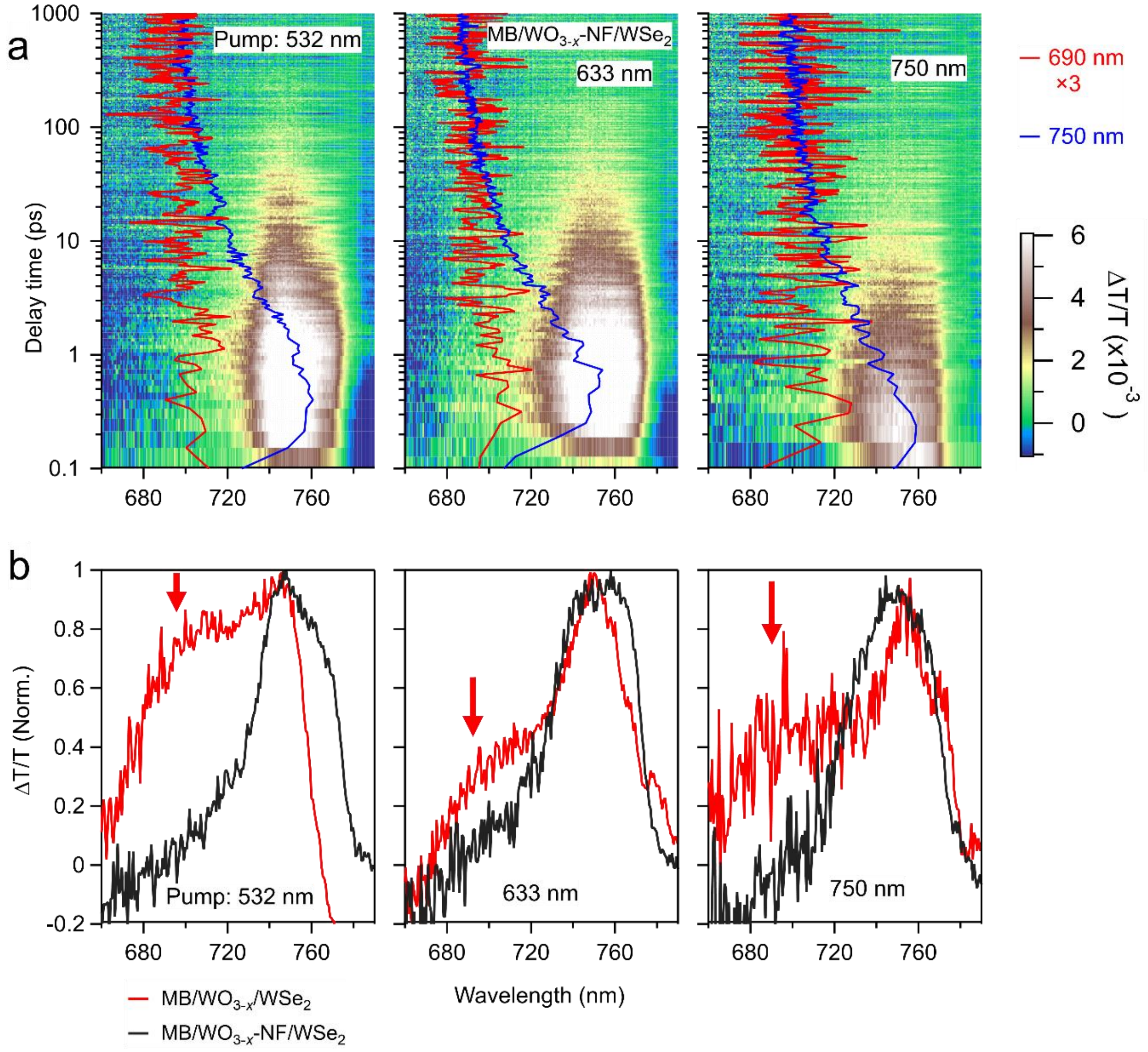

Supplementary Figure 49. Ultrafast transient transmission spectra of MB/WO_{3-x}-NF/WSe_2 and the comparison with nanowire-based MB/WO_{3-x}/WSe_2. (a) Transient transmission images of MB/WO_{3-x}-NF/WSe_2, by using different pump lights: 532 nm, 633 nm, and 750 nm. The time-dependent curves at 690 nm and 750 nm are in red and blue, respectively. (b) Wavelength-dependent data at 1.5 ps for both nanowire-based MB/WO_{3-x}/WSe_2 (red curves) and nanoflake-based MB/WO_{3-x}-NF/WSe_2 (black curves). All curves are normalized to the A exciton peak of WSe_2. The red arrows mark the MB bleaching signal.

13. Comparison between our samples and other materials as SERS substrates

Supplementary Table 1 Molecular detection performance comparison of different SERS substrates materials.

Molecules	Data source	LODs (M)	Stability (days)	Fabrication Methods	SERS substrates
MB	Our work	5×10^{-18}	28	CVD[a] at 850 °C for 6 min and Oxygen plasma at RT[b] for 2-90 s	1D/2D WO_{3-x}/WSe_2
	Ref. [12]	1×10^{-17}	-	Thermal evaporation at 250 °C for 5-10 min	Au/DFH-4T[h]
	Ref. [9]	1×10^{-14}	14	Oxidation at 680 °C for 3 min and CVD at 800 °C for 15 min	NbS_2
	Ref. [13]	8×10^{-12}	90	HF etchant for 24 h	Ti_3C_2-$Al(OH)_4$
	Ref. [14]	8×10^{-11}	-	Femtoliter surface droplets	Ag NPs-OCT[i]
	Ref. [15]	5×10^{-11}	-	Chemical reduction	Modified Lee-Meisel Ag colloid
	Ref. [16]	5×10^{-9}	-	PVD[c]	DFP-4T[j]
	Ref. [17]	1×10^{-9}	-	Ultrashort pulse laser ablation	Ag-NPs-coated LINEPSS[k]
	Ref. [18]	1×10^{-9}	-	LiF and HF etchant at 40 °C 72 h	TiVC
	Ref. [19]	1×10^{-9}	-	Liquid-phase exfoliation for 4 h and Hydrothermal method at 180 °C for 12 h	$NbTe_2$
	Ref. [20]	1×10^{-9}	-	CVD at 750 °C for 5 min and ALD[d] 250 °C for ~5 s	1T' ReS_2
	Ref. [21]	1×10^{-8}	-	CVD at 650-730 °C for 15 min	1T' $MoTe_2$
	Ref. [22]	1×10^{-7}	-	Exfoliation for 15 h and Calcination at 350 °C for 30 min	Ultrathin WO_3 nanosheet
CV	Our work	5×10^{-11}	28	CVD at 850 °C for 6 min and Oxygen plasma at RT for 2-90 s	1D/2D WO_{3-x}/WSe_2
	Ref. [19]	1×10^{-9}	-	Liquid-phase exfoliation for 4 h and Hydrothermal method at 180 °C for 12 h	$NbTe_2$
	Ref. [23]	1×10^{-8}	-	Chemical exfoliation by NaK alloy intercalation at RT for 24 h	NaK-MoS_2
	Ref. [20]	1×10^{-8}	-	CVD at 750 °C for 5 min and ALD 250 °C for ~5 s	1T' ReS_2
	Ref. [24]	1×10^{-8}	-	Carbonization at 550 °C for 5 h, KOH etchant at 80 °C and Calcination at 500 °C for 2 h, Chemical reduction	Au@H-C_3N_4
	Ref. [25]	1×10^{-8}	-	Chemical method	Cu_2O
	Ref. [26]	1×10^{-8}	60	Solvothermal polyol process and Self-	Ag@ZIF-8

				assemble method	
	Ref. [27]	2×10^{-8}	-	Chemical method	Cu_2O
	Ref. [28]	1×10^{-7}	-	PVT[e] at 850 °C for 50 h and Au-mediated exfoliation	$PdSe_2$
	Ref. [29]	2×10^{-7}	-	CVD AT 1080 °C for 5-10 min	GaN
R6G	Our work	5×10^{-10}	28	CVD at 850 °C for 6 min and Oxygen plasma at RT for 2-90 s	1D/2D WO_{3-x}/WSe_2
	Ref. [30]	1×10^{-15}	30	CVD at 1000 °C for 30 min to grow graphene and Transfer, and CVD at 500 °C for 20 min to grow ReO_xS_y	Graphene/ReO_xS_y
	Ref. [8]	4×10^{-14}	12	CVD at 800 °C for 5 min	1T' WTe_2
	Ref. [31]	1×10^{-12}	-	MBE[f] at 1100 °C for 5 min	$SnSe_2$
	Ref. [32]	5×10^{-12}	-	CVD at 1050 °C for 60 min to grow graphene and Transfer, and CVD at 450 °C for 30 min to grow MoS_2	MoS_2/Graphene
	Ref. [33]	5×10^{-9}	-	Mechanical mixer for 2 h and CVT[g] at 680 °C for 7 d	$MnPS_{3-x}Se_x$
	Ref. [34]	1×10^{-9}		CVD at 690 °C for 5-10 min	Few-layer MoS_2
	Ref. [35]	1×10^{-9}	45	Thermal evaporation and CVD at 650 °C for 4 h	1T' $MoTe_2$
	Ref. [23]	1×10^{-9}	-	Chemical exfoliation by NaK alloy intercalation at RT for 24 h	NaK-MoS_2
	Ref. [20]	1×10^{-9}	-	CVD at 750 °C for 5 min and ALD 250 °C for ~5 s	1T' ReS_2
	Ref. [19]	1×10^{-9}	-	Liquid-phase exfoliation for 4 h and Hydrothermal method at 180 °C for 12 h	$NbTe_2$
	Ref. [36]	1×10^{-9}	-	Two-step anodization, Hydrothermal at 180 °C for 6-24 h, and Electrodeposition	Pt/MoS_2/TiO_2
	Ref. [28]	1×10^{-9}	-	PVT at 850 °C for 50 h and Au-mediated exfoliation	$PdSe_2$
	Ref. [14]	1×10^{-9}	-	Femtoliter surface droplets	Ag NPs-OCT
	Ref. [25]	6×10^{-9}	-	Chemical method	Cu_2O
	Ref. [37]	1×10^{-8}	-	Liquid exfoliation for 10 h and N -butyl lithium intercalation for > 72 h	1T-$MoSe_2$
	Ref. [27]	2×10^{-8}	-	Chemical method	Cu_2O
	Ref. [10]	1×10^{-7}	-	Hydrothermal method at 200 °C for 20 h	MoS_xO_y
	Ref. [38]	1×10^{-7}	-	Hydrothermal method at 180 °C for 20 h	Metallic MoO_2
	Ref. [39]	1×10^{-7}	-	CVD at 600 °C for10 min	$SnSe_{0.93}S_{0.94}$
	Ref. [40]	1×10^{-7}	-	Hydrothermal method at 180 °C for 12 h	$W_{18}O_{49}$

[a]: *CVD denotes chemical vapor deposition*

[b]: *RT denotes room temperature*

[c]: *PVD denotes physical vapor deposition*
[d]: *ALD denotes atomic layer deposition*
[e]: *PVT denotes physical vapor transportation*
[f]: *MBE denotes molecular beam epitaxial*
[g]: *CVT denotes chemical vapor transportation*
[h]: *DFH-4T denostes α,ω-diperfluorohexylquaterthiophene*
[i]: *Ag NPs-OCT denotes Ag nanoparticles 1-octanol*
[j]: *DFP-4T: diperfluorophenyl-substituted quaterthiophene*
[k]: *LINEPSS: laser-induced nanoparticles-embedded periodic surface structures*

Supplementary References

1. Chu, X. S., Li, D. O., Green, A. A. & Wang, Q. H. Formation of MoO_3 and WO_3 nanoscrolls from MoS_2 and WS_2 with atmospheric air plasma. *J. Mater. Chem. C* **5**, 11301-11309 (2017).
2. Zhang, R., Drysdale, D., Koutsos, V. & Cheung, R. Controlled layer thinning and p-type doping of WSe_2 by vapor XeF_2. *Adv. Funct. Mater.* **27**, 1702455 (2017).
3. Wang, X. Y. et al. Noble-metal-free hybrid membranes for highly efficient hydrogen evolution. *Adv. Mater.* **29**, 1603617 (2017).
4. Liu, Y. R., Gao, Z. B., Chen, M., Tan, Y. & Chen, F. Enhanced Raman scattering of CuPc films on imperfect WSe_2 monolayer correlated to exciton and charge-transfer resonances. *Adv. Funct. Mater.* **28**, 1805710 (2018).
5. Cheng, C.-C. et al. Activating basal-plane catalytic activity of two-dimensional MoS_2 monolayer with remote hydrogen plasma. *Nano Energy* **30**, 846-852 (2016).
6. Li, H. et al. Activating and optimizing MoS_2 basal planes for hydrogen evolution through the formation of strained sulphur vacancies. *Nat. Mater.* **15**, 48-53 (2016).
7. Lin, Y.-C., Dumcenco, D. O., Huang, Y.-S. & Suenaga, K. Atomic mechanism of the semiconducting-to-metallic phase transition in single-layered MoS_2. *Nat. Nanotechnol.*

9, 391-396 (2014).

8. Tao, L. et al. 1T' transition metal telluride atomic layers for plasmon-free SERS at femtomolar levels. *J. Am. Chem. Soc.* **140**, 8696-8704 (2018).

9. Song, X. et al. Plasmon-free surface-enhanced Raman spectroscopy using metallic 2D materials. *ACS Nano* **13**, 8312-8319 (2019).

10. Zheng, Z. H. et al. Semiconductor SERS enhancement enabled by oxygen incorporation. *Nat. Commun.* **8**, 1993 (2017).

11. Zhou, J. D. et al. A library of atomically thin metal chalcogenides. *Nature* **556**, 355-359 (2018).

12. Yilmaz, M. et al. Nanostructured organic semiconductor films for molecular detection with surface-enhanced Raman spectroscopy. *Nat. Mater.* **16**, 918-924 (2017).

13. Li, G. et al. Surface-modified two-dimensional titanium carbide sheets for intrinsic vibrational signal-retained surface-enhanced Raman scattering with ultrahigh uniformity. *ACS Appl. Mater. Interfaces* **12**, 23523-23531 (2020).

14. Li, M. S., Dyett, B., Yu, H. T., Bansal, V. & Zhang, X. H. Functional femtoliter droplets for ultrafast nanoextraction and supersensitive online microanalysis. *Small* **15**, 1804683 (2019).

15. Anastasopoulos, J. A., Soto Beobide, A., Manikas, A. C. & Voyiatzis, G. A. Quantitative surface-enhanced resonance Raman scattering analysis of methylene blue using silver colloid. *J. Raman Spectrosc.* **48**, 1762-1770 (2017).

16. Demirel, G. et al. Molecular engineering of organic semiconductors enables noble metal-comparable SERS enhancement and sensitivity. *Nat. Commun.* **10**, 5502 (2019).

17. Hamad, S. et al. Femtosecond laser-induced, nanoparticle-embedded periodic surface structures on crystalline silicon for reproducible and multi-utility SERS platforms. *ACS Omega* **3**, 18420-18432 (2018).

18. He, Z. et al. Two-dimensional TiVC solid-solution MXene as surface-enhanced Raman scattering substrate. *ACS Nano* **16**, 4072-4083 (2022).

19. Wang, K. et al. Few-layer $NbTe_2$ nanosheets as substrates for surface-enhanced Raman scattering analysis. *ACS Appl. Nano Mater.* **3**, 11363-11371 (2020).

20. Miao, P. et al. Unraveling the Raman enhancement mechanism on 1T'-phase ReS_2 nanosheets. *Small* **14**, 1704079 (2018).

21. Adhikari, B., Limbu, T. B., Vinodgopal, K. & Yan, F. Atmospheric-pressure CVD growth of two-dimensional 2H- and 1 T'-$MoTe_2$ films with high-performance SERS activity. *Nanotechnology* **32,** 335701 (2021).

22. Song, G., Gong, W. B., Cong, S. & Zhao, Z. G. Ultrathin two-dimensional nanostructures: Surface defects for morphology-driven enhanced semiconductor SERS. *Angew. Chem. Int. Ed* **133**, 5565-5571 (2021).

23. Er, E. et al. High-yield preparation of exfoliated 1T-MoS_2 with SERS activity. *Chem. Mater.* **31**, 5725-5734 (2019).

24. Tang, W., An, Y. & Ho Row, K. Fabrication of Au nanoparticles embedded holey g-C_3N_4 as SERS substrates for sensitive and reliable detection. *Chem. Eng. J.* **402**, 126194 (2020).

25. Lin, J. et al. Ultrasensitive SERS detection by defect engineering on single Cu_2O superstructure particle. *Adv. Mater.* **29**, 1604797 (2017).

26. Li, Q. et al. Tailored necklace-like Ag@ZIF-8 core/shell heterostructure nanowires for high-performance plasmonic SERS detection. *Chem. Eng. J.* **371**, 26-33 (2019).

27. Li, X. et al. Temperature-induced stacking to create Cu_2O concave sphere for light trapping capable of ultrasensitive single-particle surface-enhanced Raman scattering. *Adv. Funct. Mater.* **28**, 1801868 (2018).

28. Lei, Z. et al. Enhanced Raman scattering on two-dimensional palladium diselenide. *Nanoscale* **14**, 4181-4187 (2022).

29. Zhao, S. et al. 2D GaN for highly reproducible surface enhanced Raman scattering. *Small* **17**, 2103442 (2021).

30. Seo, J. et al. Ultrasensitive plasmon-free surface-enhanced Raman spectroscopy with femtomolar detection limit from 2D van der Waals heterostructure. *Nano Lett.* **20**, 1620-1630 (2020).

31. Li, W. et al. Tunable 3D light trapping architectures based on self-assembled $SnSe_2$ nanoplate arrays for ultrasensitive SERS detection. *J. Mater. Chem C* **7**, 10179-10186 (2019).

32. Ghopry, S. A., Alamri, M. A., Goul, R., Sakidja, R. & Wu, J. Z. Extraordinary sensitivity of surface-enhanced Raman spectroscopy of molecules on MoS_2 (WS_2) nanodomes/graphene van der Waals heterostructure substrates. *Adv. Optical Mater.* **7**, 1801249 (2019).

33. Hou, X. Y. et al. Alloy engineering in few-layer manganese phosphorus trichalcogenides for surface-enhanced Raman scattering. *Adv. Funct. Mater.* **30**, 1910171 (2020).

34. Majee, B. P., Mishra, S., Pandey, R. K., Prakash, R. & Mishra, A. K. Multifunctional

few-layer MoS_2 for photodetection and surface-enhanced Raman spectroscopy application with ultrasensitive and repeatable detectability. *J. Phys. Chem. C* **123**, 18071-18078 (2019).

35. Fraser, J. P. et al. Application of a 2D molybdenum telluride in SERS detection of biorelevant molecules. *ACS Appl. Mater. Interfaces* **12**, 47774-47783 (2020).

36. Dong, J. et al. Vertically-aligned Pt-decorated MoS_2 nanosheets coated on TiO_2 nanotube arrays enable high-efficiency solar-light energy utilization for photocatalysis and self-cleaning SERS devices. *Nano Energy* **71**, 104579 (2020).

37. Yin, Y. et al. Significantly increased Raman enhancement on MoX_2 (X = S, Se) monolayers upon phase transition. *Adv. Funct. Mater.* **27**, 1606694 (2017).

38. Zhang, Q. et al. A metallic molybdenum dioxide with high stability for surface enhanced Raman spectroscopy. *Nat. Commun.* **8**, 14903 (2017).

39. Tian, Y. et al. Influence of SERS activity of $SnSe_2$ nanosheets doped with sulfur. *Nanomaterials* **10,** 1910 (2020).

40. Cong, S. et al. Noble metal-comparable SERS enhancement from semiconducting metal oxides by making oxygen vacancies. *Nat. Commun.* **6**, 7800 (2015).
